# An evaluation of sample size requirements for developing risk prediction models with binary outcomes

**DOI:** 10.1186/s12874-024-02268-5

**Published:** 2024-07-10

**Authors:** Menelaos Pavlou, Gareth Ambler, Chen Qu, Shaun R. Seaman, Ian R. White, Rumana Z. Omar

**Affiliations:** 1 Department of Statistical Science, UCL, London, UK; 2 MRC Biostatistics Unit, University of Cambridge, Cambridge, UK; 3 MRC Clinical Trials Unit at UCL, London, UK

**Keywords:** Sample size, Simulation, Calibration, Discrimination

## Abstract

**Background:**

Risk prediction models are routinely used to assist in clinical decision making. A small sample size for model development can compromise model performance when the model is applied to new patients. For binary outcomes, the calibration slope (CS) and the mean absolute prediction error (MAPE) are two key measures on which sample size calculations for the development of risk models have been based. CS quantifies the degree of model overfitting while MAPE assesses the accuracy of individual predictions.

**Methods:**

Recently, two formulae were proposed to calculate the sample size required, given anticipated features of the development data such as the outcome prevalence and c-statistic, to ensure that the expectation of the CS and MAPE (over repeated samples) in models fitted using MLE will meet prespecified target values. In this article, we use a simulation study to evaluate the performance of these formulae.

**Results:**

We found that both formulae work reasonably well when the anticipated model strength is not too high (c-statistic < 0.8), regardless of the outcome prevalence. However, for higher model strengths the CS formula underestimates the sample size substantially. For example, for c-statistic = 0.85 and 0.9, the sample size needed to be increased by at least 50% and 100%, respectively, to meet the target expected CS. On the other hand, the MAPE formula tends to overestimate the sample size for high model strengths. These conclusions were more pronounced for higher prevalence than for lower prevalence. Similar results were drawn when the outcome was time to event with censoring. Given these findings, we propose a simulation-based approach, implemented in the new R package ‘samplesizedev’, to correctly estimate the sample size even for high model strengths. The software can also calculate the variability in CS and MAPE, thus allowing for assessment of model stability.

**Conclusions:**

The calibration and MAPE formulae suggest sample sizes that are generally appropriate for use when the model strength is not too high. However, they tend to be biased for higher model strengths, which are not uncommon in clinical risk prediction studies. On those occasions, our proposed adjustments to the sample size calculations will be relevant.

**Supplementary Information:**

The online version contains supplementary material available at 10.1186/s12874-024-02268-5.

## Introduction

Clinical prediction models are routinely used in practice for prognosis or diagnosis. They can provide individual predictions given patient characteristics and may allow both clinicians and patients to monitor the course of a disease and make informed decisions regarding clinical management. For example, the QRISK prediction model [1] has been incorporated into clinical practice as a tool to estimate the 10-year risk of cardiovascular disease, guiding lifestyle changes and the need for preventative treatment. Another example is the HCM-SCD risk model [2] which is used to estimate the risk of Sudden Cardiac Death (SCD) in patients with hypertrophic cardiomyopathy (HCM).

Prediction models are often derived using regression models although other approaches including machine learning methods may be used [3]. These model the association between an outcome variable and a set of explanatory variables. For binary outcomes, such as in-hospital mortality, a logistic regression model is often used. The model coefficients are estimated using development (training) data and this model may then be used to make predictions for new patients. The predictive ability of the model is typically assessed using either the development dataset via data-splitting, bootstrapping or cross-validation, or a validation (test) dataset [4]. If this model shows satisfactory performance with respect to calibration, discrimination and overall predictive accuracy, the model can be recommended for use in practice. It is important that the sample size of both the development and validation datasets are sufficient. In particular, if the development dataset is too small, the resulting model may fit the development data too well (overfitting) and predict poorly in validation data.

Therefore, there is a need for clear guidelines regarding the sample size requirements for developing a reliable risk model. Until recently, the ‘rule of 10’ was often used which suggests that at least 10 events per predictor variable (EPV) are required for developing risk models [5, 6]. Recently, though, van Smeden et al. [7] performed a simulation study to investigate the effect of various factors on risk model performance, including EPV, model discrimination (see subsection ‘Model Performance’), outcome prevalence, and number and type of predictors. They concluded that predictive accuracy depends on sample size, number of predictors and outcome prevalence, and provided several formulae to calculate the sample size needed to achieve a desired level of predictive accuracy. Riley et al. [8]. derived different sample size formulae based on either controlling the degree of model overfitting or estimating the prevalence of the outcome accurately (overall risk). The conclusions and sample size formulae (hereafter RvS) from these two papers are summarised in a joint paper by Riley et al. [9]. This contains four sample size formulae for binary outcomes based on: (i) estimation of overall risk; (ii) estimation of individual risk; (iii) controlling overfitting; (iv) controlling optimism in apparent model fit. The recommended sample size is the largest number obtained across all four formulae.

In this paper, we investigate the performance of two of these sample size formulae, specifically those based on the estimation of individual risk and controlling overfitting, since they concern aspects that are typically among the most important in model development. Furthermore, in practice, the two formulae we investigate most often produce the largest of the four sample sizes. We therefore first investigate whether each of these performs as intended and then investigate how often they lead to risk models that have ‘acceptable’ performance, where we define acceptable performance in terms of model calibration and discrimination.

In our main simulation study, we investigate the RvS formulae for binary outcomes, varying model strength and outcome prevalence with weakly correlated predictor variables. We then perform additional simulations to investigate the sensitivity of the results to the degree of correlation between continuous predictors, the type of predictors (continuous or binary) and the type of outcome (binary or time to event). We found that the RvS sample size formulae were biased in some scenarios, and so we develop unbiased simulation-based sample size calculations and implement these in the R package ‘samplesizedev’ (available from the github repository https://github.com/mpavlou/samplesizedev).

This paper is organised as follows. In the ‘Methods’ section we describe the methods typically used to develop and validate risk models for binary outcomes and the RvS sample size formulae. In the ‘Simulations’ section we describe simulation studies to assess the performance of RvS formulae. Given the findings of the simulation study we then present a simulation-based approach to calculate the sample size for binary outcomes. The final section is a discussion.

## Methods

### Prediction models for binary outcomes

Prediction models for binary outcomes are commonly developed using logistic regression. The model

$$\pi =\text{Pr}\left(Y=1|\varvec{x}\right)= \frac{1}{1+\text{exp}\left(-\eta \right)}$$

models the probability ($\pi$) of an event as a function of the linear predictor $\eta ={\beta }_{0}+{\beta }_{1}{x}_{1}+\dots {\beta }_{p}{x}_{p}={\varvec{\beta }}^{\varvec{T}}\varvec{x}$, where ${\beta }_{j}$ and ${x}_{j}$ are the regression coefficient and predictor value for the j-th predictor and Y is the binary outcome. Estimation of the regression coefficients is typically performed using maximum likelihood estimation (MLE); these estimates can then be used to make predictions for new patients. Prediction models are often developed in a ‘development’ dataset then tested using a separate ‘validation’ dataset, where model performance is typically evaluated in terms of calibration, discrimination and predictive accuracy (the accuracy of individual predictions) [10].

### Model performance

Two common measures for assessing the predictive performance of risk models are the calibration slope and c-statistic which, respectively, quantify the agreement between observed and predicted risks and the concordance between the predictions and outcomes (measuring discrimination). In addition, one might calculate the mean absolute prediction error (MAPE) to quantify the distance between the estimated and ‘true’ probabilities (measuring predictive accuracy) [7]. We note that MAPE can only be calculated when we know the true probabilities, i.e., in simulation.

In detail, calibration may be assessed by considering the relationship between the outcomes and the predictions using a logistic regression model [4, 11]. In detail, the following logistic model (calibration model) is fitted to validation data of size ${n}_{val}$

$$\text{log}\left(\frac{{\pi }_{i}}{1-{\pi }_{i}}\right)={\alpha }_{0}+{\alpha }_{1}{\widehat{\eta }}_{i}, i=1,\dots , {n}_{val}$$

where ${\widehat{\eta }}_{i}$ is the estimated linear predictor, calculated using regression coefficients estimated in the development data of size $n$. Parameter ${\alpha }_{1}$ is known as the calibration slope (CS), with values less than 1 suggestive of model overfitting. The calibration model above can also be used in internal validation (e.g. cross-validation and bootstrap validation).

The c-statistic (also known as the area under the ROC curve) is the probability that a patient who has an event has a higher predicted risk than a patient who does not have an event. This can be estimated using

$$c = {{\sum\nolimits_{i = 1}^{{n_{{\rm{val }}}}} {\sum\nolimits_{j = 1}^{{n_{{\rm{val }}}}} I } \left( {{y_i} = 1\;\& \;{y_j} = 0} \right)\left\{ {I\left( {{{\hat \pi }_i} > {{\hat \pi }_j}} \right) + 0.5I\left( {{{\hat \pi }_i} = {{\hat \pi }_j}} \right)} \right\}} \over {\sum\nolimits_{i = 1}^{{n_{{\rm{val }}}}} {\sum\nolimits_{j = 1}^{{n_{{\rm{val }}}}} {I\left( {{y_i} = 1\;\& \;{y_j} = 0} \right)} } }}$$

where $$\widehat{\pi }={\left\{1+\text{exp}\left(-\widehat{\eta }\right)\right\}}^{-1}$$$$\text{a}\text{n}\text{d} \ I\left(u\right)$$ equals 1 if $$u$$ is true and 0 otherwise.

The mean absolute prediction error (MAPE) is the mean absolute difference between the estimated and true probabilities. This may be estimated using

$$MAPE=\frac{1}{{n}_{val}}\sum _{i=1}^{{n}_{val}}\left|{\widehat{\pi }}_{i}-{\pi }_{i}\right|.$$

We might also determine whether the performance of a risk model is acceptably close to that of the true model. We assume that the performance of the fitted model is assessed in a dataset with the same characteristics as the original development dataset (i.e. the development and validation dataset are random samples from the same population). For example, for calibration, we may consider performance to be unacceptable if the calculated calibration slope is less than 0.8. For discrimination, we may consider performance to be acceptable if the estimated c-statistic is within 0.02 of the true c-statistic. We use these definitions later in our simulations.

### Shrinkage

Logistic regression models estimated using MLE tend to exhibit some degree of overfitting [12, 13]. That is, the highest predictions tend to be too high and the lowest too low [4]. As discussed earlier, the degree of overfitting may be quantified using the CS.

In practice, shrinkage is often used to counteract overfitting [4]. One simple approach is to estimate and apply a shrinkage factor $S$ to the coefficient estimates following MLE. That is, the prediction model becomes

$$\text{log}\left(\frac{\widehat{\pi }}{1-\widehat{\pi }}\right)={\widehat{\beta }}_{0}^{*}+S\left({\widehat{\beta }}_{1}{x}_{1}+\dots {\widehat{\beta }}_{p}{x}_{p}\right)$$

where the intercept ${\beta }_{0}^{*}$ is re-estimated so that the average predicted probability equals the outcome prevalence. This has the effect of shrinking the individual predictions towards the overall outcome prevalence, and, on average should result in a calibration slope close to one in validation data.

The ‘heuristic’ shrinkage factor may be calculated as

1 $$S=\left({\Delta }{\chi }^{2}-p\right)/{\Delta }{\chi }^{2}$$

where ${\Delta }{\chi }^{2}$ is the model deviance and $p$ is the number of model parameters (excluding the intercept) [14]. As noted by Van Houwelingen & Le Cessie (1990) [15], this relationship should be valid if the model strength (c-statistic) is modest and the predictor variables follow a multivariable normal distribution.

A shrinkage factor may also be estimated using the bootstrap. Briefly, the model is fitted in bootstrap datasets with the original dataset used for validation. The average value of the calibration slope over these bootstraps is an estimate of the shrinkage factor. Finally, shrinkage may also be applied at the estimation stage, for example using a penalised regression method such Ridge or Lasso [16, 17]. We do not consider penalised regression methods further in this work, since the sample size formulae that are the focus of our evaluation assume that the models are fitted using MLE.

### Formulae for the Sample size of the Development Sample

RvS describe four separate sample size formulae and recommend choosing the maximum value obtained from these. We investigate the performance of two of these formulae and describe these below.

This first of these formulae (hereafter RvS-1 or ‘calibration formula’) is based on controlling model overfitting and is derived using the equation for the heuristic shrinkage factor [15]. Riley et al. (2019) [8] show that the sample size *n* needed to achieve a target expected shrinkage factor of *S* (hereafter ‘target expected shrinkage’ or ‘target expected CS’ for conciseness) after MLE has been used for model fitting is given by

$$n=\frac{p}{\left(S-1\right)\text{log}\left(1-\frac{{R}_{CS}^{2}}{S}\right)}, \quad \text { (RvS - 1)}$$

where ${R}_{CS}^{2}$ is the Cox-Snell ${R}^{2}$ statistic (proportion of variance explained), a measure of model strength, and $p$ is the number of model parameters (excluding the intercept). In line with [8], throughout this paper we assume that variable selection is not performed. We note here that RvS-1 depends on the model strength and the outcome prevalence via ${R}_{CS}^{2}$. RvS suggest that the chosen value of $S$ be no lower than 0.9. The expected shrinkage, or ‘expected calibration slope’, $S,$ is interpreted to mean that if the model were to be fitted to many random samples of size $n$ from the population of interest and validated on infinitely large validation datasets from the same population, then the calculated CS would be on *average*, $S.$

The second equation that we investigate (hereafter RvS-2 or ‘MAPE formula’) calculates the sample size for estimating individual predictions accurately and was derived from the simulation results of van Smeden et al. (2018) [7]. The sample size *n* needed to achieve a target expected mean absolute prediction error (*MAPE*) $m$ is given by

$$n=\exp \left(\frac{-0.508+0.259 \log (\phi)+0.504 \log (p)-\log (m)}{0.544}\right), \quad \text { (RvS - 2)}$$

where $\phi$ is the anticipated outcome prevalence. RvS-2 does not consider model strength in the calculation. RvS recommend that *m* be no larger than 0.05, though, in practice, this choice should arguably depend on the prevalence of the outcome. Without loss of generality, we later use $m=\phi /10$ in our simulations when evaluating formula RvS-2, although in practice $m$ can be set to any value deemed appropriate. The target expected MAPE is interpreted in an analogous way to the target expected CS.

For completeness, we mention that the two other formulae provide the sample size for estimating the mean predicted risk (e.g., to within 0.05), and for controlling the optimism in the estimate of the Nagelkerke ${R}^{2}$ statistic. The latter is another measure of model strength, and optimism is defined as the difference between the apparent model performance, as quantified in the development data, and the actual model performance, as quantified in validation data. We do not consider these formulae further for the reasons stated in the introduction.

## Simulations

### Design

Simulation studies were used to investigate the performance of the RvS-1 (calibration) and RvS-2 (MAPE) sample size formulae. The RvS-1 formula was derived by Riley et al. [8] using the equation for the heuristic shrinkage factor, which assumes modest discrimination in the data [14]. It is therefore important to assess the magnitude and direction of possible bias of RvS-1 when model strength is high (i.e., whether using the sample size suggested by RvS-1 results in the target expected CS). The RvS-2 formula was derived by van Smeden et al. [7] using simulation, and model strength is not included as part of the equation. Therefore, it is of interest to assess its validity for a range of model strengths. Based on these motivations, we considered different scenarios corresponding to different combinations of model strength (c-statistic) and outcome prevalence. We note that higher of values of ${R}_{CS}^{2}$ (of which Nagelkerke’s ${R}^{2}$ is a function) and the c-statistic both correspond to a greater predictive ability for a model (higher model strength). As values of ${R}_{CS}^{2}$ are rarely reported in the literature [18], we chose to define model strength in terms of the c-statistic in our simulation results. We describe these simulations below using the ADEMP framework of Morris et al. (2019) [19].

#### Aims

The primary aim of the simulations was to investigate whether the sample sizes selected by the RvS formulae led to risk models with the anticipated performance for different combinations of prevalence and model strength. In detail, we investigated whether choosing the sample size using RvS-1 and RvS-2 resulted in fitted models with the target expected CS and MAPE, respectively (i.e., whether the mean CS equals the target expected CS, and similarly for MAPE).

For RvS-1 we also investigated the variability in the CS (quantified by the root mean square distance of the calibration slope – see ‘Performance measures’ section below) and calculated the probability of obtaining a model with unacceptable calibration (defined here as $CS<0.80$) and a c-statistic close (within 0.02) to the true value.

#### Data-generating mechanisms

For each scenario we generated 2000 development and validation datasets each containing the binary outcome and 12 predictor variables; five of these were true predictors (${\beta }_{j}\ne 0$) and seven were noise variables (${\beta }_{j}=0$), following Riley 2021 [18]. The predictor variables were generated from a multivariate normal distribution with mean zero and unit variance, with pairwise correlations of 0.1 between the true predictors, 0.05 between the noise predictors, and 0 between noise and true predictors. The binary outcomes were generated using the Bernoulli distribution with parameter $\pi$, where $\pi =logi{t}^{-1}\left({\varvec{\beta }}^{T}\varvec{x}\right)$ and $\varvec{\beta }$ and $\varvec{x}$ denote the vector of regression coefficients and predictor values respectively.

The size of the development datasets for each scenario were determined using either RvS-1 with target $S=0.9$, or RvS-2 with target expected MAPE $m=\phi /10$. For RvS-1, we calculated ${R}_{CS}^{2}$ after fitting a model to a very large dataset with one million observations. Alternatively, ${R}_{CS}^{2}$ can be approximated using the c-statistic, assuming a normal distribution for the linear predictor in patients with and without the event [20]; in the simulation we report results from using the true ${R}_{CS}^{2}.$ Similarly, the value of the c-statistic for the true model, which we call ‘true c-statistic’ for conciseness, was obtained by calculating the c-statistic in the same validation dataset using the true probabilities $\pi$.

The validation datasets were generated using the same data generating mechanism, but with 100,000 observations. The large size of the validation datasets ensures that the values of the performance metrics (see below) for the fitted model are estimated with very little variability.

The values of the regression coefficients were chosen to correspond to a desired outcome prevalence $\phi$ and model strength scenario. Specifically, we set $\varvec{\beta }=\left({\beta }_{0}, f\times \varvec{\gamma }\right)$, with $\varvec{\gamma }=\left(0.4, 0.2, 0.2, 0.1, 0.1, 0, 0, 0, 0, 0, 0, 0\right)$ denoting the relative strength of the predictors, and chose ${\beta }_{0}$ and $f$ accordingly to match the required prevalence and c-statistic.

#### Targets

We focus on measures of predictive performance when models are estimated using datasets with sample sizes obtained using formulae RvS-1 or RvS-2. We consider the CS, the MAPE and the c-statistic.

#### Parameter values

Six values of model strength (c-statistic = 0.65, 0.70, 0.75, 0.80, 0.85 $\text{and}$ 0.90) and three values of outcome prevalence (10%, 30% and 50%) were investigated. The sample sizes indicated by the RvS formulae are shown in Table 1; for each sample size $n$, the EPV was calculated as $EPV=n \phi /p$.

**Table 1:** Calculated sample size (n - rounded to the nearest 10) and corresponding EPV using the calibration (RvS-1) and MAPE (RvS-2) formulae in Riley et al. (9)

Prevalence	C-statistic	*n* RvS-1	EPV RvS-1	*n* RvS-2	EPV RvS-2
0.1	0.65	4120	34.3	6230	51.9
0.3	0.65	1780	44.6	1400	34.9
0.5	0.65	1480	61.7	700	29.0
0.1	0.75	1390	11.6	6230	51.9
0.3	0.75	620	15.5	1400	34.9
0.5	0.75	520	21.8	700	29.0
0.1	0.85	640	5.3	6230	51.9
0.3	0.85	290	7.2	1400	34.9
0.5	0.85	250	10.2	700	29.0

#### Methods

We performed the simulations as follows for each combination of outcome prevalence and model strength. First, we generated 2000 development datasets with sample sizes determined as described above. We then fitted logistic regression models to the development datasets using MLE and calculated the measures of predictive performance (CS, MAPE and c-statistic) using the validation datasets. The use of 2000 simulations for each scenario ensured that the Monte Carlo simulation error (MCSE) was sufficiently small; the maximum value of the MCSE across all scenarios was 0.003 for the calibration slope, 0.0002 for the c-statistic and 0.0004 for MAPE.

#### Performance measures

For each scenario, we assessed the performance of the sample size formulae RvS-1 and RvS-2 by comparing the mean calculated calibration slope and MAPE values to their target values, 0.9 and $\phi /10$, respectively.

One issue with the CS is its variability. Even when the mean CS appears to be close to the target expected CS, it tends to exhibit very high variability in some scenarios [18, 21]. Consequently, we looked at the Root Mean Square Distance of the CS (RMSD) from the ideal value of 1, which has been suggested [21] as a suitable measure to assess model performance with respect to CS. In addition, to further assess variability in model performance, we also calculated the proportion of times the estimated model exhibited unacceptable calibration ($CS< 0.8$ suggesting substantial overfitting) or acceptable discrimination (c-statistic for the estimated model within 0.02 of the true c-statistic).

Whenever the target expected CS and MAPE were not achieved with the recommended sample sizes using formulae RvS-1 and RvS-2, we also obtained by simulation the sample sizes *actually required* to achieve the target values on average.

To calculate the required sample size we used the bisection method (which requires provision of starting values for the sample size and re-simulation and calculation of CS and MAPE until they are, on average, close enough to the target expected values). More details on our proposal for simulation-based sample size calculations are in a following section and in the Supplementary Material 1 (section ‘Details for simulation-based sample size calculations’). The software code (R) used for the main simulation study is provided in the Supplementary Material 2.

## Results

### Calibration slope

**Fig. 1:**
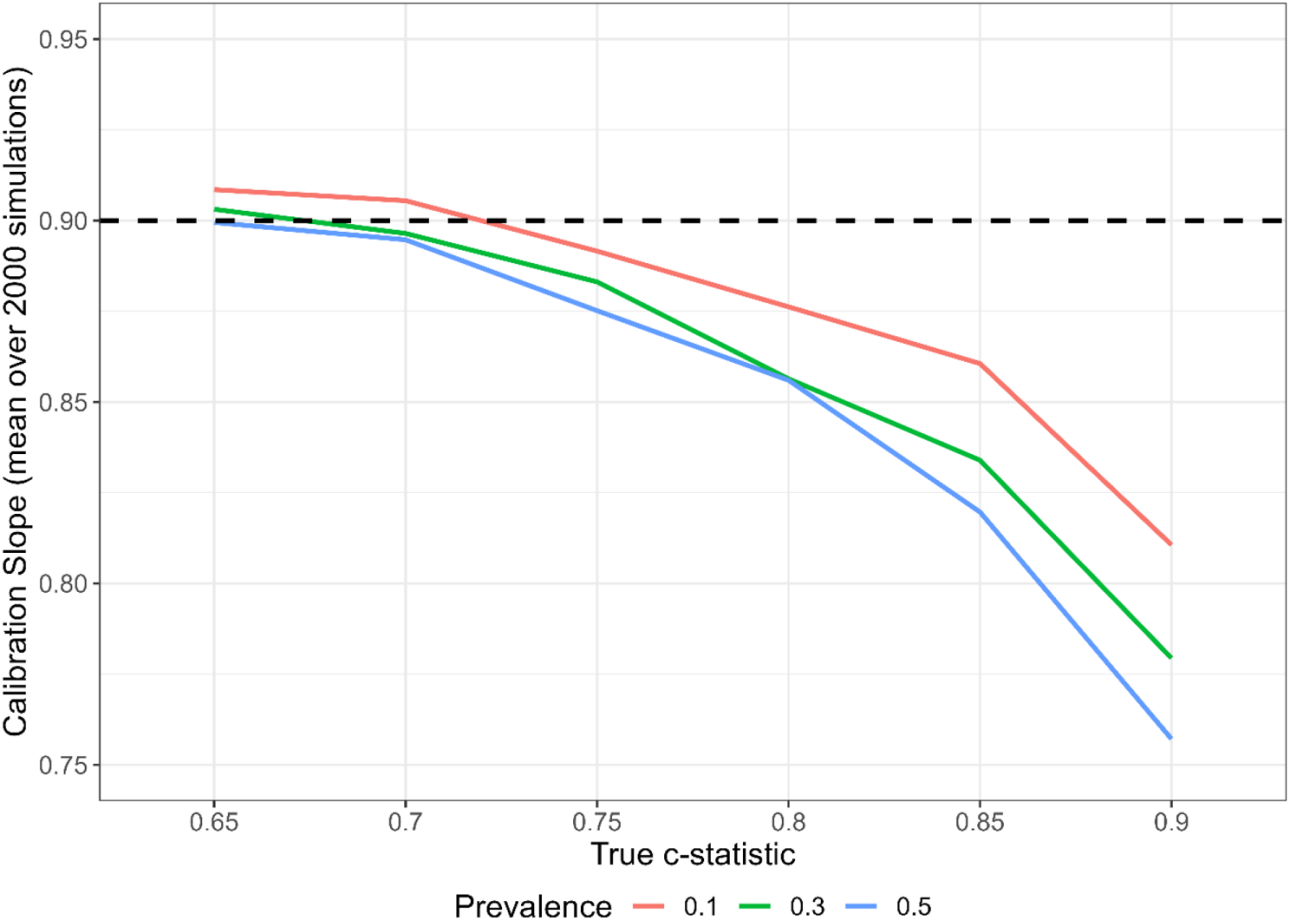
Mean calibration slope for different values of model strength and outcome prevalence, using the sample size calculated using the RvS-1 calibration formula with target expected CS of $S=0.90$. Based on 2000 simulations

Figure 1 shows the mean CS for models developed with sample sizes calculated using RvS-1. If the RvS-1 formula worked well, then all the lines would lie near the horizontal dotted line. The target expected CS is $S=0.9$ for all combinations of model strength (c-statistic) and outcome prevalence. We see that the performance of RvS-1 depends on model strength and, to a lesser degree, outcome prevalence. That is, the mean CS is close to 0.90 when the model strength is relatively low but diverges from it as model strength increases. When the c-statistic is 0.90, the mean CS is 0.82 or less, depending on outcome prevalence. The CS also worsens with increasing prevalence. Figure S1 (figures prefixed by ‘S’ are in the Supplementary Material 1) shows that the variability in the CS tends to increase with model strength (primarily due to the under-estimation of the sample size).

**Fig. 2:**
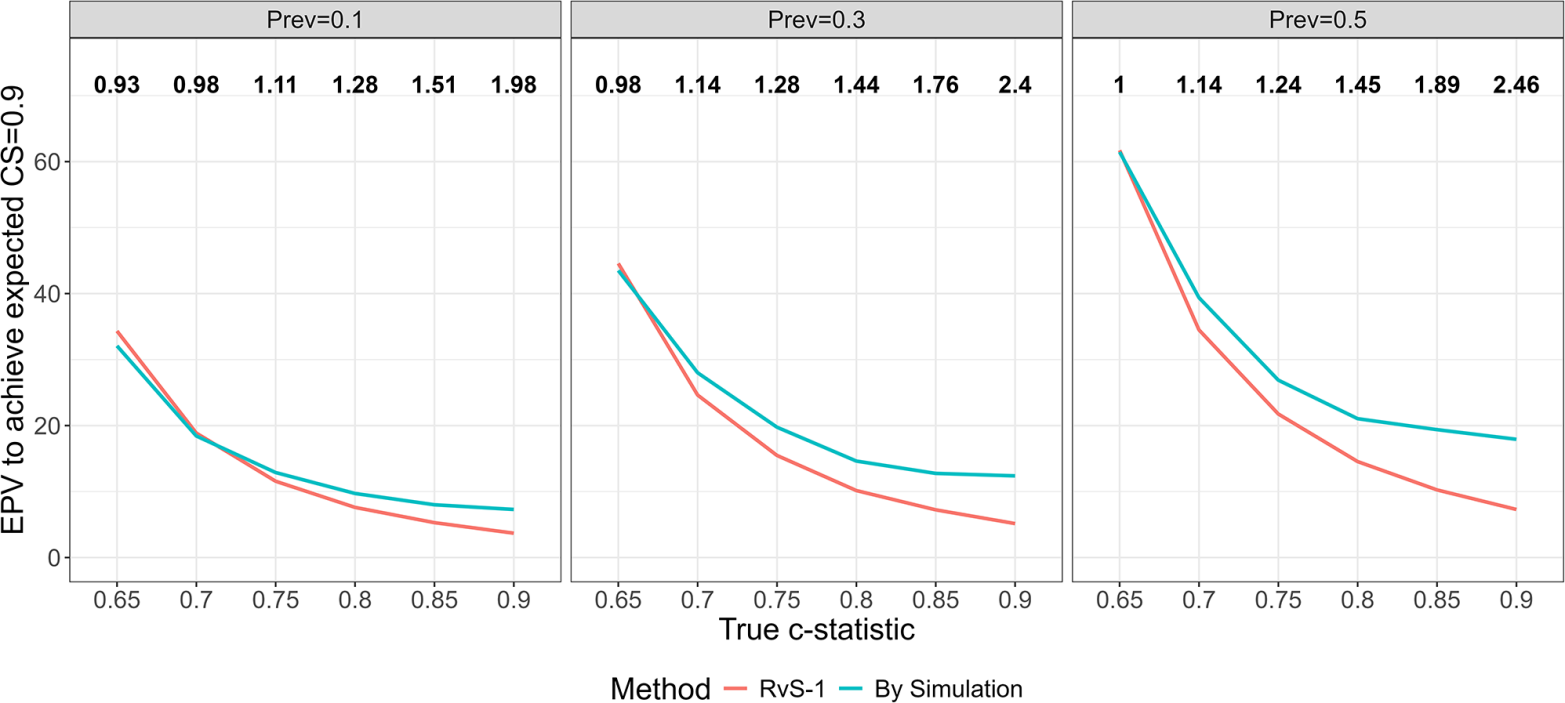
The EPV required to achieve target expected CS of $S=0.90$ calculated by simulation (blue line) and using the RvS-1 calibration formula (red line) for different values of model strength and outcome prevalence (Prev). Numbers on top correspond to the ratio of the EPV calculated by simulation to the EPV calculated using RvS-1. Based on 2000 simulations

Figure 2 shows, using RVS-1 and using simulation, the sample size required to achieve the target expected CS, for different values of model strength and outcome prevalence. We express sample size via EPV to enable comparisons with the rule of 10 and across different scenarios. As in Fig. 1, it is clear that much larger sample sizes than that suggested by RvS-1 are required for higher values of model strength ($c\ge 0.8$). This is particularly so for higher values of outcome prevalence. For example, when c-statistic=0.85 and prevalence=0.1, an EPV of 8 is required compared to the RvS-1 value of 5.3. If the prevalence is 0.5, then an EPV of 19.4 is required compared to the RvS-1 value of 10.2. Further investigation suggests that the reason why RvS-1 is less accurate when model strength is high is that the heuristic shrinkage factor Eq. (1) under-estimates the amount of shrinkage that is required in these scenarios (results not shown). The recommended EPV using equation RvS-1 and the EPV calculated by simulation to achieve the target expected CS are provided in Table S1. Finally, we note that when ${R}_{CS}^{2}$ was approximated using the c-statistic, the sample sizes obtained by the RvS-1 formula were very close to the sample sizes obtained using the true ${R}_{CS}^{2}$, and hence the conclusions were the same.

**Fig. 3:**
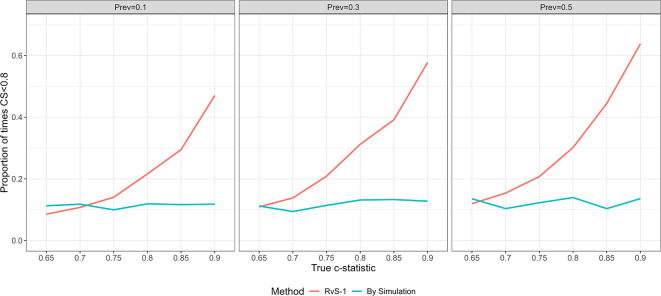
The proportion of simulations with CS < 0.8 for different values of model strength and outcome prevalence, using: (a) the sample size calculated using the RvS-1 calibration formula with target expected CS of $S=0.90$ (left) and (b) the sample size calculated by simulation to achieve the target expected CS (right). Based on 2000 simulations

Figure 3 shows the proportion of models with $CS<0.8$. When the sample size is chosen using RvS-1, the probability of obtaining a model with $CS<0.8$ ranges from around 0.1 for low model strengths to 0.6 for high model strength. When the sample size is correctly chosen via simulation to achieve the target expected CS of $S=0.9$, the probability is reasonably constant at around 0.12.

Figure S2 shows the proportion of models with acceptable discrimination, that is, a c-statistic for the estimated model within 0.02 of the true c-statistic. We can see that use of RvS-1 tends to produce a model with discrimination somewhat below the true value for higher model strengths. In contrast, when the sample size is correctly chosen to achieve the target expected CS, most models have discrimination close to the true value across all model strengths.

### MAPE

**Fig. 4:**
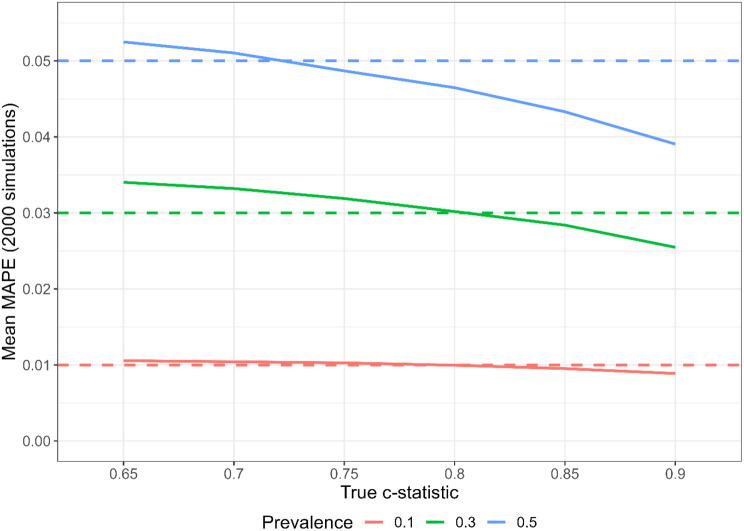
Mean MAPE for different values of model strength and outcome prevalence, using the sample size calculated using the RvS-2 MAPE formula with target MAPE $m=\text{prevalence}/10$. Based on 2000 simulations. Dashed lines show the three target expected MAPEs for the three prevalences

Figure 4 shows the average MAPE (Figure S3 shows the variability in MAPE) for models developed using sample sizes calculated using RvS-2. The target value of expected MAPE is $\phi /10$ for all combinations of model strength and outcome prevalence $\phi$. The performance of RvS-2 seems to depend on both model strength and outcome prevalence. More specifically, the mean MAPE typically exceeds the target value slightly when the model strength is low but decreases below the target value as model strength increases. This trend is more evident for higher values of outcome prevalence.

**Fig. 5:**
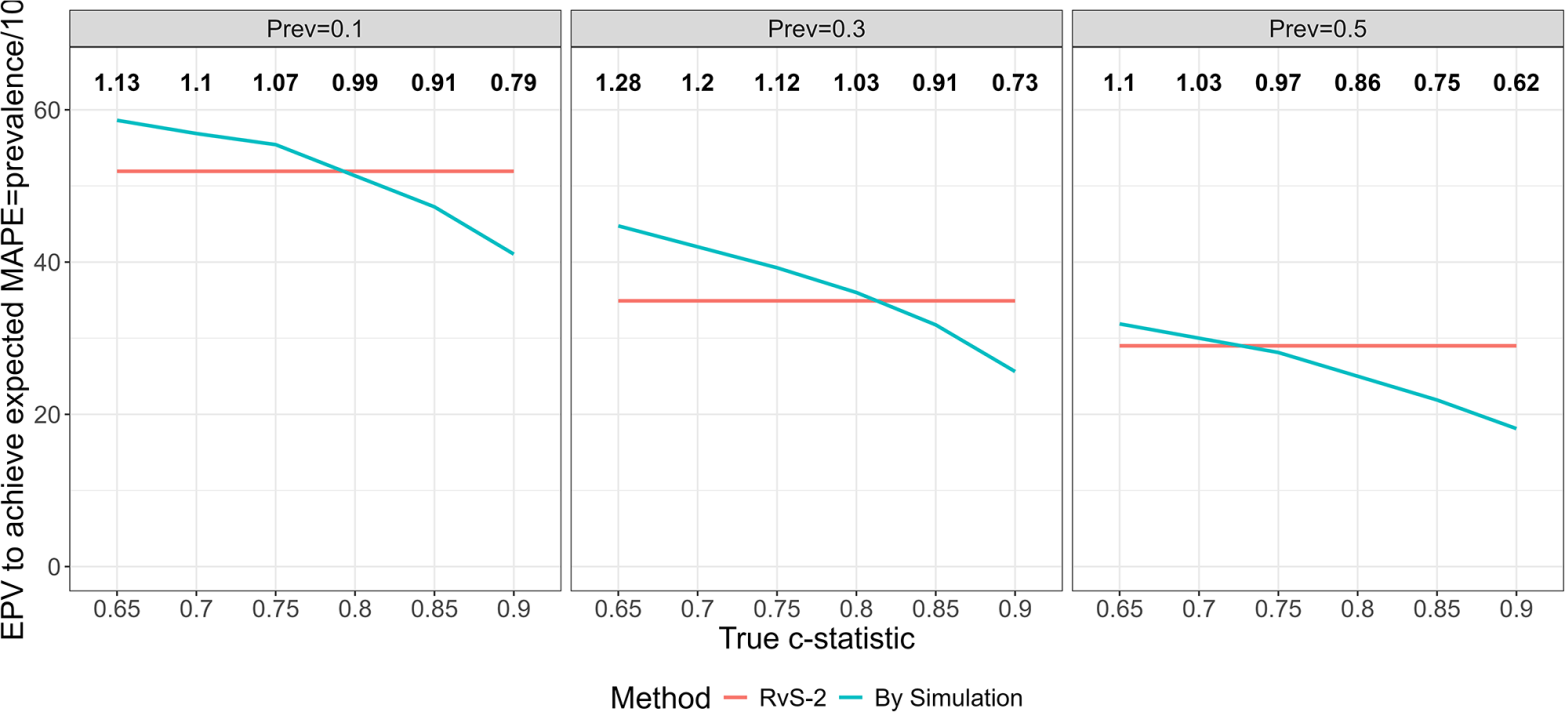
The EPV required to achieve the target $MAPE=\text{prevalence}/10$ calculated by simulation (blue line) and using the RvS-2 MAPE equation (red line) for different values of model strength and prevalence. Numbers on top correspond to the ratio of the EPV calculated by simulation to the EPV calculated using RvS-2. Based on 2000 simulations

Figure 5 shows the sample size calculated by simulation, expressed via EPV, needed to achieve the target MAPE for different values of model strength and outcome prevalence. It is clear that smaller sample sizes could be used in many circumstances, particularly for higher values of model strength. For low values of model strength, a slightly larger sample size might be required. For example, when the c-statistic and prevalence are 0.85 and 0.1 respectively, an EPV of 47.2 is required compared to the RvS-2 value of 51.9. If prevalence is 0.5, then an EPV of 21.9 is required compared to the RvS-2 value of 29. The recommended EPV using RvS-2 and the EPV calculated by simulation to achieve the target *MAPE* are shown in Table S2.

### Further analyses

We performed additional simulation studies, analogous to those described in the previous section, to assess the sensitivity of the results to: (i) correlations between continuous predictor variables; (ii) binary predictors; (iii) number of predictor variables, iv) different type of outcome (time to event).

#### Correlation between continuous predictors

We first calculated the sample size using either the RvS formulae or simulation assuming the same correlations between predictors (weakly correlated) and the same relative strength of predictors as in the main simulation. We then modified the part of the DGM that concerns the generation of the predictor variables. Specifically, we generated continuous predictors, either uncorrelated or correlated, and selected the regression coefficients to correspond to an outcome prevalence of 0.1 and model strengths ranging from 0.65 to 0.85. For correlated predictors, the correlation between the continuous true predictors was set to either 0.5 or 0.8, and the correlation between the noise predictors was set to 0.3.

For the chosen size, we calculated the mean calibration slope and MAPE in datasets where the true correlations between the predictors differed, as above. We found that the conclusions of the previous section remained unchanged for both RvS-1 and RvS-2 formulae (Table S3). Also, for a given size the mean calibration slope and MAPE were very similar regardless of the degree of correlation between the predictors.

#### Binary predictors

We then considered a model with only independent binary predictors with prevalences ranging between 0.2 and 0.7. This covariate pattern resulted in a relatively skewed linear predictor. The mean calibration slope and MAPE were very similar to the case of continuous and correlated predictors (Table S3). These results suggest that, for given values of the c-statistic and prevalence and a given sample given size, the expected CS and MAPE do not seem to vary substantially depending on the type of covariates and correlation between covariates, at least for the scenarios considered here.

#### Number of predictor variables

We then studied whether the number of predictor variables ($p$) affect the performance of the formulae. For this evaluation, we assumed independent and normally distributed predictor variables of equal strength. We considered a low model strength scenario (c-statistic = 0.7), for which RvS-1 formula was seen to work well (see the previous section for $p=12$). The target expected CS was chosen to be $S=0.9$ as earlier, the anticipated outcome prevalence was fixed to 0.1 and $p$ was varied between 4 and 30. The mean CS was overall very close to the target value of 0.9 (Figure S4). However, a notable finding was that the variability in the CS and hence, the RMSD of the CS was much higher when $p$ was less than 10. This can be explained by the fact that the required sample size decreased for smaller $p$. As a result, the probability of obtaining a miscalibrated model was much higher for smaller $p$ than for larger $p.$ For, instance the chance of obtaining a model with $CS<0.8$ was 21% when $p=4$, and only 8% when the $p=22.$ This suggests that care should be taken when the number of predictor variables is small. Ideally, the target expected CS should be chosen so as the probability of obtaining a severely miscalibrated model is low. The results for MAPE were analogous (not shown).

#### Time to event outcome with censoring

We then considered whether the conclusions for equation RvS-1 hold when the outcome is time to event. We modified the part of the DGM that concerns the outcome, to generate time to event outcomes with censoring from the proportional hazards model $h\left(t\right)={h}_{0}\left(t\right)\text{exp}\left({\beta }^{T}\varvec{x}\right)$, where $h\left(t\right)$ is the hazard function at time $t$ and ${h}_{0}\left(t\right)$ is the baseline hazard function. We specified a constant baseline hazard and hence, survival times were generated using the exponential distribution. We considered uncorrelated normally distributed predictors (5 true and 7 noise as in the main simulation study). We quantified the model strength using the concordance or Harrell’s c-index [22] (considering two patients, c-index is the probability that the patient with the largest value of the linear predictor has the shortest survival time). The variance of the normally distributed linear predictor was chosen to match a desired concordance, analogously to the c-statistic for binary outcomes. We administratively censored the survival times at a particular time-point to ensure that the proportion of uncensored observations matched a prespecified value (0.1, 0.5, 0.9).

The results (shown in Table S4) were similar to those for binary outcomes when the proportion of censored individuals was 0.5 or higher (proportion of events up to 0.5). The similarity was perhaps to be expected because the corresponding RvS-1 equation for time to event outcomes is derived using the same shrinkage factor Eq. (1) as that used to derive the binary version of RvS-1. When using RvS-1, the sample size was appropriate for low and medium-strength models but was underestimated for higher strength models. Underestimation was worse when there was less censoring.

## Simulation-based sample size calculations to achieve target expected calibration slope and MAPE for binary outcomes

We now describe the approach briefly mentioned in the previous section (and used for Figs. 2 and 5) that uses simulation and optimisation to calculate the sample size required to achieve a target expected CS or MAPE for binary outcomes. This approach is computationally efficient and has been implemented in the R package samplesizedev (available from the github repository https://github.com/mpavlou/samplesizedev). Full details can be found in Supplementary Material 1 (Box 1 and Box 2 in Section ‘Details for simulation-based sample size calculations’).

The software requires the following inputs: anticipated values of the *outcome prevalence*, the *c-statistic* and the *number of predictor variables.*

It can either:

- calculate the sample size *if the user inputs a target value for the expected CS or MAPE*.
- calculate the expected CS and MAPE (and also the variability in these measures which enables assessment of model stability) *if the user inputs a sample size.*

The sample size calculation is based on the assumption that the predictor variables follow a multivariate normal distribution, which is also the assumption underpinning formula RVS-1. We also make the simplifying assumption that the predictors are independent. As seen in our simulation study (subsection ‘Further analyses’), provided that the linear predictor is chosen to have mean and variance to match the anticipated prevalence and c-statistic, the correlation between the predictor variables minimally affects the expected CS and MAPE for a given sample size. The independence assumption is helpful for two reasons. First, it simplifies the level of input required by the user, and second, it allows us to perform some of the computations using algebra and numerical integration [23, 24], which is faster than using simulation. These calculations and our full algorithm for simulation-based sample size calculations are provided in the Supplementary Material 1.

We have observed that the MCSE will be sufficiently small (for the CS the MCSE will usually be less than 0.0025 at the calculated size to achieve a target expected CS of $S=0.9$) when we use at least ${n}_{sim}=1000$ simulated development datasets, and validation datasets of size at least ${n}_{val}$=25,000. Indicatively, for ${n}_{sim}=1000$ and ${n}_{val}=25,000,$ the routine usually takes around one minute to complete.

### Example

Suppose that we wish to develop a risk model with 24 predictor variables and the anticipated prevalence and c-statistic are $\phi =0.174 \text{ and } c=0.89,$ respectively. These are the input parameters example provided in the R package pmsampsize [25] and discussed in [8]. Using formula RvS-1, the required sample size to achieve a target expected CS of $S=0.9$ is 620 (rounded up to the nearest 10).

We use the package samplesizedev to evaluate whether this sample size is adequate to meet the calibration target. All results below were obtained assuming 24 predictors of equal strength; the results were almost identical when we used different numbers of true/noise predictors and relative strengths (the code and detailed results are provided in the Supplementary Material 1).

**Fig. 6:**
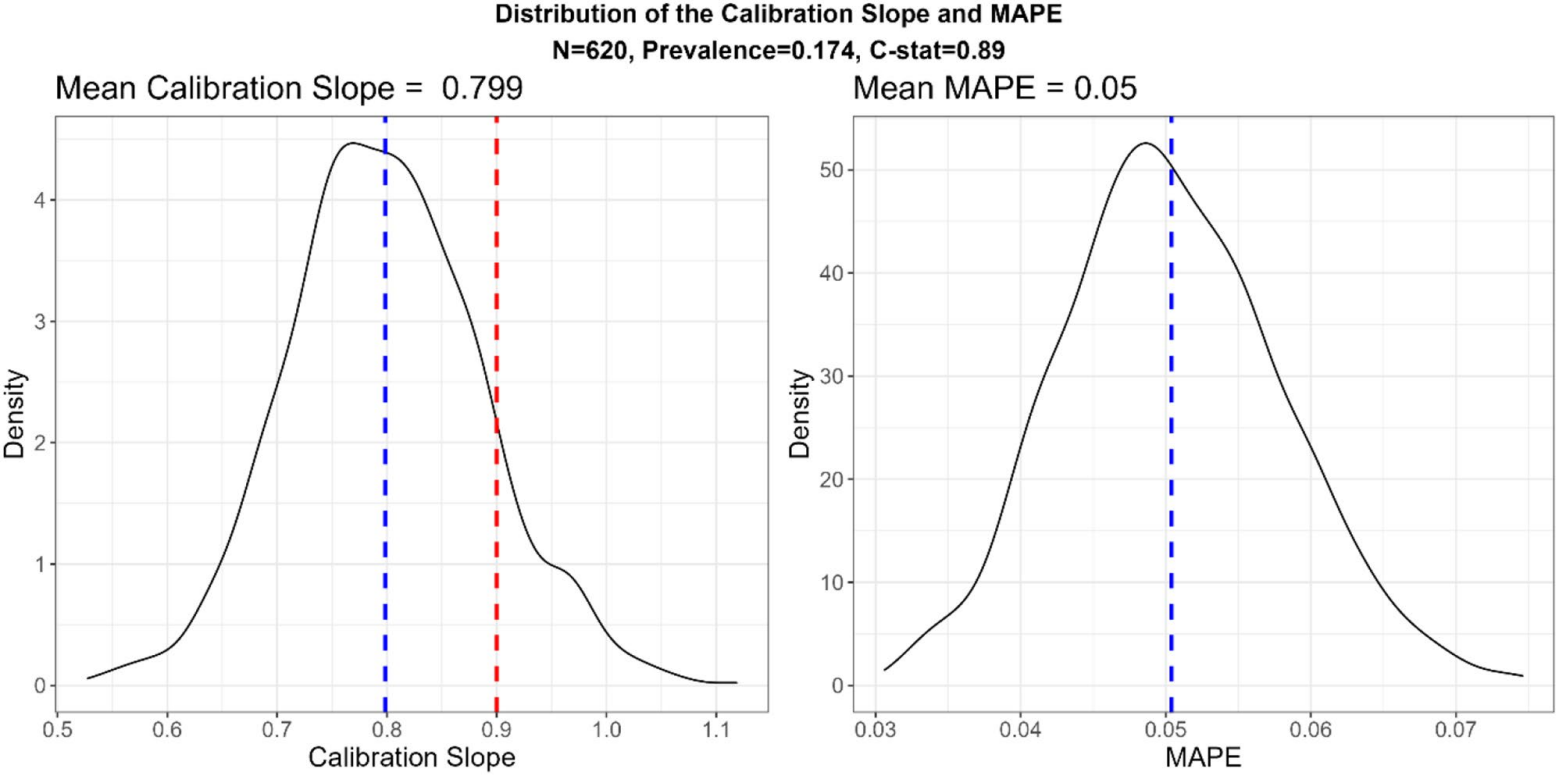
The distribution of the calibration slope and MAPE for the recommended sample size of the development sample based on RvS-1 calibration formula

In line with the simulation results in the previous section, the sample size is substantially underestimated by RvS-1. For the recommended sample size of 620, the mean CS is 0.80 $(MCSE=0.0027),$ well below the target expected calibration slope of $0.9$. For this sample size, the variability in the CS is substantial (Fig. 6) and the probability of obtaining a model with CS below 0.9 and 0.8 is very high, around 86% and 52%, respectively. Using simulation with the package samplesizedev, the required size to achieve the expected CS of $S=0.9$ is *more than double*, 1310.

Similarly, using equation RvS-2, the recommended sample size to achieve expected MAPE $m=0.05$ is 800. For this recommended size, the mean MAPE is slightly lower than 0.05, indicating a slight overestimation of the sample size. Using simulation, the required sample size to achieve a target expected MAPE of $m=0.05$ is 630.

### Advantages and limitations of the simulation-based approach

The advantages of our proposed simulation-based sample size calculations compared to the existing calculations are: (1) unbiased estimation of the sample size even for high model strengths and (2) estimation of the variability in the measures of predictive performance, which allows for assessment of model stability. A disadvantage is that by using our software, it may take a minute (for each of CS and MAPE) to calculate the sample size which, although not prohibitively slow, is slower than using the RvS software.

It is worth noting that the simulation-based approach to sample size calculation was primarily used to assess the RvS formulae under ideal conditions (where the c-statistic, outcome prevalence and number of predictor variables are considered known, and the predictor variables are normally distributed). Although, it can be adapted to more complex scenarios, its application in practice will be challenging because the additional information required to simulate from those scenarios may not be readily available before data collection. For example, if we were to assume that the distribution of the linear predictor is non-normal, we would require information regarding the distribution and relative strength of the individual predictors, a level of information that would usually not be available before data collection. In our sensitivity analyses (section ‘Further analyses’), we did not observe substantial variation in the expected CS and MAPE (for a given sample size), with different types of predictor variables and different levels of correlation between these variables, but further future investigations are warranted.

## Discussion

We have used simulation to investigate the performance of the sample size formulae proposed by Riley and van Smeden for the development of risk prediction models for binary outcomes. Specifically, we investigated the performance of the calibration and mean absolute prediction error (MAPE) formulae for different values of model strength (c-statistic) and outcome prevalence.

The results from the first set of simulations suggest that the calibration equation (RvS-1) works well when the model strength is low to moderate but tends to severely under-estimate the sample size requirements when the model strength is high (c-statistic > 0.8). This suggests the sample size calculated using RvS-1 may need to be increased in such scenarios. For example, we observed that depending on the prevalence, the sample size needed to be increased by at least 20%, 50%, and 100% when the c-statistic was 0.8, 0.85 and 0.9, respectively. Our simulations suggest that ensuring that the expected CS is at least 0.9, the resulting model will also have a high chance of achieving acceptable discrimination, defined here as achieving a c-statistic within 0.02 of the true c-statistic.

The results from the second set of simulations, in contrast, suggest that the MAPE equation (RvS-2) may over-estimate the sample size requirements when the model strength is high. This suggests that a smaller sample size might be adequate in such scenarios though we would generally recommend a conservative approach.

In a series of further analyses, we investigated whether the results above hold when the model includes correlated (continuous) predictors or binary predictors, when the number of predictors varies, or when a time to event outcome (with censoring) is used. For both formulae we found that the results were very similar in the presence of correlated predictors or binary predictors. When varying the number of predictor variables for model strength equal to 0.7, a scenario where we had previously seen RVS-1 and RVS-2 working well, we found that that the performance target (CS/MAPE) was still met on average. Nevertheless, the variability was particularly high when the number of predictor variables was smaller than 10. Finally, as expected, the results for RvS-1 were also similar when applied to a time to event outcome with proportion of censoring 50% or higher. For lower censoring proportions, the performance of RvS-1 was worse for time to event than that for binary outcome.

Overall, the RvS calibration and MAPE formulae suggest sample sizes that are generally appropriate for use in practice when the model strength is not too high (c-statistic < 0.8). Certainly, they are more nuanced than those suggested by the old ‘rule of 10’, which do not change depending on important factors such as model strength. However, it is not uncommon to observe a c-statistic > 0.8 in clinical risk prediction studies [26]. Arguably, higher values of the c-statistic (e.g. > 0.8) may be more common in diagnostic models than in prognostic models and hence, care should be taken when using RvS formulae in those cases. Information regarding the anticipated value for the c-statistic and outcome prevalence can often be obtained from existing risk models, as described in detail in [8]. In the absence of reliable information, we suggest choosing a conservative value for the anticipated value of the c-statistic to avoid obtaining a sample size that is too small.

In this paper we have thoroughly evaluated the two main formulae from RvS (calibration and MAPE formulae). These typically produce the largest sample sizes of the four formulae proposed and hence, in practice, will often determine the chosen sample size. Regarding the two formulae that were not evaluated in detail, we note the following. The formula based on the optimism in Nagelgerke’s $${R}^{2}$$ ($${R}_{Nag}^{2})$$ is obtained using the same approximations used for the calibration formula. To calculate the sample size to meet a target expected optimism $$\delta$$ in $${R}_{Nag}^{2}$$, the corresponding target shrinkage $${S}_{\delta }$$ is first calculated. Then, the required sample size is obtained by plugging $${S}_{\delta }$$ into the calibration formula. The formula to ensure the precise estimation of overall risk makes the key assumption that the risk for an individual with mean predictor values (which is obtained as the inverse logit of the intercept $${\beta }_{0}$$ in a model where all predictors have been mean-centred) will often be very similar to the mean risk in the overall population ($$\phi$$). While this statement may hold when the discrimination (c-statistic) is small, it does not hold in general, with large deviations when the prevalence is smaller than 0.5 and the c-statistic is moderate to high. For example, when $$\phi =0.1$$ and $$c=0.75\text{ and } 0.8, $$$${logi{t}^{-1}(\beta }_{0})$$ will be equal to $$0.072 \text{ and } 0.058$$, respectively (assuming a normally distributed linear predictor). Hence, the estimand $${logi{t}^{-1}(\beta }_{0})$$ does not, in general, correspond to a quantity we might be interested in, and so the related sample size formula for precise estimation of $${logi{t}^{-1}(\beta }_{0})$$ seems of limited practical use.

In practice, it is important that the sample size be chosen with the clinical aims of the model in mind. The RvS formulae investigated in this paper are important because they consider two important aspects of predictive performance: calibration and predictive accuracy. However, they only target average values of calibration slope and MAPE and there is, of course, no guarantee that an individual model fitted on an adequately sized sample from the target population will achieve these values. Even in cases where a calibration target is met on *average*, the variability in the calibration slope can be quite high. One such scenario we have seen in this article is when the number of candidate predictor variables is smaller than 10. Our simulation-based approach, implemented in the software ‘samplesizedev’, in addition to estimating the sample size required to achieve a target calibration slope on average, also allows quantification of the *variability* in the calibration slope for that sample size.

### Electronic supplementary material

Below is the link to the electronic supplementary material.

Supplementary Material 1

Supplementary Material 2

## Data Availability

In this study we used synthetic (simulated data) for method evaluation. Software code (R) written for the simulation studies is available from the Supplementary Material 2.

## Abbreviations

CS: Calibration Slope
EPV: Events Per Variable
MAPE: Mean Absolute Prediction Error
MCSE: Monte Carlo Simulation Error
MLE: Maximum Likelihood Estimation
HCM: Hypertrophic cardiomyopathy
RMSD: Root Mean Square Distance
RvS: Riley – van Smeden formulae
SCD: Sudden Cardiac Death
