# Supplementary material for: An evaluation of sample size requirements for developing risk prediction models with binary outcomes
Source: BMC Med Res Methodol. 2024 Jul 10;24:146. doi: 10.1186/s12874-024-02268-5 (PMC11234534; doi:10.1186/s12874-024-02268-5)

Supplementary Material 1

# Details for simulation-based sample size calculations for binary outcomes

## Calculation of expected performance for a given size

**Box 1**

**Step E1:** *Specify values for the anticipated outcome prevalence (*$\phi$*), the c-statistic (c), the joint distribution of the* $k$ *predictor variables,* ***X****, and a vector* $\boldsymbol{\gamma}$ *for the relative strength of the predictor variables such that* $\sum{|\gamma}_{k}|=1.$

Example: in our main simulation, $k=12, \gamma=(0.4, 0.2, 0.2,0.1,0.1, 0, \ldots,0 )$ where $\beta_{k}=f\times\gamma_{k}$ and $\beta_{0}$ is the intercept term. Also, $X\sim MVN\left( 0, \Sigma\right)$ where $\Sigma$ is a block diagonal matrix with the correlation between the true variables being 0.1 and between noise 0.05. Using simulation and optimisation (for example with the optim function in R) we can find $\beta_{0}$ and $f$ such that the outcome prevalence and c-statistic and $p$ and $c$ respectively.

**Step E2:** *Generate development and validation datasets*

For a given size, $n_{dev},$ for the development data we generate $n_{sim}$ sets of covariates from the assumed distribution $(X_{k}$ $k=1,\ldots p$) and outcomes $\left( Y\sim\mathrm{Bernoulli}\left( \mathrm{logi}t^{-1}\left( \beta_{0}+\sum_{k} {\beta_{k}X}_{k} \right) \right) \right)$ with the chosen $\boldsymbol{\beta}$from Step E1. Aso, generate large validation datasets of size $n_{val}$ using the same data generating model.

**Step E3:** *Calculate measures of predictive performance.*

We fit a logistic regression model to each of the $n_{sim}$ development datasets using MLE to obtain $\hat{\boldsymbol{\beta}}$. We calculate $\hat{\eta}=\hat{\beta}_{0}+\sum_{k} {\hat{\beta}_{k}X}_{k}$ and $\hat{\pi}=logit^{-1}\left( \hat{\eta} \right)$ in the validation data and calculate measures of predictive performance.

In our main simulation we calculate the calibration slope, MAPE, and the c-statistic. The expected shrinkage and MAPE are the corresponding means over the $n_{sim}$ values. The probability of obtaining a model with unacceptable calibration or acceptable discrimination are the proportion of times the calibration slope is less than 0.8 and the proportion of times the estimated c-statistic is within 0.02 of the true value.

**Alternative step E1, assuming independent and Normally distributed Predictor variables**

Under the assumption that the predictor variables are independent and normally distributed, simulation can be avoided in Step E1; instead numerical integration can be used which makes the process faster. Specifically, Step E1 can be replaced by steps E1A and E1B below.

**Step E1A:**

*Specify anticipated outcome prevalence (*$\phi$*) and c-statistic (c). Find the values for the mean and variance of an underlying normally distributed linear predictor that corresponds to* $\phi$ *and* $c.$

We assume that the linear predictor is a normally distributed variable $\eta\sim N\left( \mu, \sigma^{2} \right)$ with cumulative distribution function $F.$ The binary outcome is $Y\sim Bernoulli\left( logit^{-1}\left( \eta\right) \right).$We aim to find $\mu$ and $\sigma$ that correspond to the anticipated prevalence $\phi=P\left( Y=1 \right)$ and (true) c-statistic, $c$ of the risk model. For given values of $\mu$ and $\sigma^{2}$ , $\phi$and $c$ may be calculated using numerical integration.[[17](#_ENREF_17), [18](#_ENREF_18)]

For the outcome prevalence

|  | $\phi=P\left( Y=1 \right)=\int_{-\infty}^{+\infty} \pi(\eta) dF\left( \eta\right).$ | (1) |
| --- | --- | --- |

where $\pi\left( x \right)=logit^{-1}\left( x \right).$

For the c-statistic

$c=P\left( \eta^{\left( 1 \right)}>\eta^{\left( 0 \right)} \right)$ where $\eta^{\left( 1 \right)}$ = $\eta|Y=1$ and $\eta^{\left( 0 \right)}=$ $\eta|Y=0$ with cumulative distribution functions $P\left( \eta^{\left( 1 \right)}\leq x \right)=\frac{\int_{-\infty}^{x} \pi\left( \eta\right)\mathrm{dF}\left( \eta\right)}{\int_{-\infty}^{\infty} \pi\left( \eta\right)\mathrm{dF}\left( \eta\right)}$ and $P\left( \eta^{\left( 0 \right)}\leq x \right)=\frac{\int_{-\infty}^{x} \left( 1-\pi\left( \eta\right) \right)\mathrm{dF}\left( \eta\right)}{\int_{-\infty}^{\infty} \left( 1-\pi\left( \eta\right) \right)\mathrm{dF}\left( \eta\right)} ,$respectively.

Consequently,

$$c =\frac{\int_{-\infty}^{+\infty} P\left( \eta^{\left( 1 \right)}>w \right)(1-\pi\left( w \right)\mathrm{dF}(w)}{\int_{-\infty}^{+\infty} (1-\pi\left( w \right)\mathrm{dF}(w)} which gives$$

|  | $c= \frac{\int_{-\infty}^{+\infty} ( \int_{-\infty}^{w} \pi\left( z \right) dF(z)) \left( 1-\pi\left( w \right) \right) dF(w)}{\int_{-\infty}^{+\infty} \int_{-\infty}^{+\infty} \pi\left( z \right)(1-\pi\left( w \right)\mathrm{dF}\left( z \right) dF(w)}$ | (2) |
| --- | --- | --- |

where $w,z\sim N\left( \mu, \sigma^{2} \right)$. As there is no analytical solution for $\mu$ and $\sigma^{2}$ in terms of $\phi$ and $c$ in (1) and (2) above, we obtain the solution numerically using optimisation (for example with the optim function in R). This only takes a few seconds.

**Step E1B:** *Specify number (and relative strength) of predictors* $p$ *to obtain the vector of regression coefficients.*

Let $X_{k}\overset{iid}{\sim}N\left( 0, 1 \right),$ $k=1,\ldots p$ and let $\boldsymbol{\gamma}$ denote the vector of their relative strength such that $\sum_{k} |\gamma_{k}|=1.$ The linear predictor is then $\eta=\mu+\sum_{k} {\beta_{k}X}_{k}$ where$\beta_{k}=f\times\gamma_{k}$, and $var\left( \eta\right)=\sum_{k} \beta_{k}^{2}=f^{2}\sum_{k} {\gamma_{k}}^{2}$.

Parameter $\mu$ is known from Step 1. We calculate $f$ such that $var\left( \eta\right)=\sigma^{2}$ which gives

$f=\sqrt{\frac{\sigma^{2}}{\sum_{k} {\gamma_{k}}^{2}}} .$ For example, $\gamma_{k}=\frac{1}{p}\forall k$ means that all predictors are of equal strength and $f=\sigma\sqrt{p}.$ The selected values of $\mu$ and $\boldsymbol{\beta}$ ensure that the anticipated outcome prevalence is $\phi$ and the c-statistic for the true model is $c.$

Step E1, E1A, E1B, E2 and E3 have been implemented in the R package ‘samplesizedev’with the command: expected_cs.

Our investigations showed use of $n_{sim}\geq1000$ with $n_{val}\geq25000$ ensures that the Monte Carlo Simulation Error (MCSE) will be sufficiently small. In our simulations we used $n_{sim}=2000$ and $n_{val}=100,000$ which led to the maximum values of the MCSE being 0.0003 for calibration slope and 0.0002 for MAPE.

## Calculation of sample size to meet a target performance

We describe below the calculation for the required sample size to meet a target expected CS (and analogously for MAPE), assuming normally distributed linear predictors.

**Box 2**

**Inputs: Anticipated features of the data and target expected calibration slope**

Outcome prevalence : $\phi$

C-statistic : $c$

Number of predictors : $p$

Relative strength of predictors: $\boldsymbol{\gamma}$

Target Expected CS : $S_{targ}$

**Outputs**

The required sample size : $n_{req}$

**Process**

**S1:** Given $\phi$, $c$ and $p$ run Steps E1or E1A and E1B

**S2:** Provide lower ($n_{min}$) and upper ($n_{max}$) starting values for the sample size. Reasonable values are suitable multiples of the sample sizes of Rvs-1 ($n_{RvS-1}$). For example, for the expected CS, based on the results of the simulation studies we may choose $n_{min}=0.5\times n_{RvS-1}$ and $n_{max}=4\times n_{RvS-1}.$

**S3:** Run Steps E3-E4 for $n_{min}$ and $n_{max}$ to obtain $S_{min}$ and $S_{max}$, respectively.

**S4:** If |$S_{min}-S_{targ}|<\mathrm{tolerance}$ $n_{req}=n_{min}$,

otherwise if ${|S}_{max}-S_{targ}|<\mathrm{tolerance}$ $n_{req}=n_{max}$,

otherwise if $|S_{min}-S_{targ}|$ < ${|S}_{max}-S_{targ}|$

set: $n_{min}=n_{min} , n_{max}= \frac{n_{min}+ n_{max}}{2}$ and go to Step S3

otherwise set: $n_{min}= \frac{n_{min}+ n_{max}}{2} , n_{max}=n_{max}$ and go to Step S3

The tolerance is set to equal to 0.0025 for CS and (target MAPE)/200 for MAPE. These values will be larger than the Monte Carlo Simulation error when $n_{sim}\geq1000$ and $n_{val}\geq25000.$

## Examples using the software ‘samplesizedev’

Suppose we want to calculate the sample size required to achieve an expected calibration slope of 0.9, when there are 24 predictors, and anticipated values foe the true outcome prevalence and the c-statistic is p=0.174 and 0.89, respectively.

First install the package using:

require(devtools)

devtools::install_github("mpavlou/samplesizedev")

require(samplesizedev)

The calculated size when using the RVS-1 calculation to achieve target expected CS of S=0.9 is 620 (round up to the nearest 10). According, to the results of our simulation study, this sample size must be too small to achieve the expected calibration slope because the c-statistic is quite high. We can check whether this calculation is adequate using simulation and the package samplesizedev.

# Assuming predictors of equal strength (default and assumed for RVS-1)

expected_cs(n = 620, phi = 0.174, c = 0.89, p = 24, nsim = 1000)

N Mean_CS SD_CS Pr(CS<0.8) Mean_MAPE SD_MAPE

620 **0.799** 0.0881 0.52 0.0499 0.0076

# Assuming predictors of different relative strength $\boldsymbol{\gamma=}(0.4, 0.2, 0.1, 0.1, 0.05, 0.05, 0, 0,\ldots0\boldsymbol{)}$

gamma <- c(0.4, 0.2, 0.1, 0.1, 0.05, 0.05, rep(0,18))

expected_cs(n = 620, phi = 0.174, c = 0.89, p = 24, nsim = 1000, gamma=gamma)

N Mean_CS SD_CS Pr(CS<0.8) Mean_MAPE SD_MAPE

620 **0.800** 0.0862 0.5 0.0507 0.0075

# Assuming predictors of different relative strengths, $\boldsymbol{\gamma=}(0.8, 0.2, 0, 0,\ldots0\boldsymbol{)}$

gamma <- c(0.8, 0.2, rep(0,22))

expected_cs(n = 620, phi = 0.174, c = 0.89, p = 24, nsim = 1000, gamma=gamma)

N Mean_CS SD_CS Pr(CS<0.8) Mean_MAPE SD_MAPE

620 **0.803** 0.0886 0.5 0.0505 0.0078

R*egardless of the chosen relative strength of predictors*, the results are almost identical, indicating that the sample size is underestimated by RVS-1, as the mean CS is substantially lower than the target value of 0.9. In fact, there is 50% that with this size the developed model will have a calibration slope less than 0.8, which is unacceptably low. We note that the sample size based on the criterion for obtaining optimism in Nagelkerke’s $R^{2}$ δ=0.05 was 661, which corresponds to a - marginally better - expected calibration slope of 0.81. The sample size based on the formula for obtaining a precise calculation of the overall risk was only 251.

Using the package samplesizedev we can calculate the correct size to achieve an expected calibration slope of 0.9, on average (assuming 24 predictors of equal strength)

samplesizedev(S = 0.9, phi = 0.174, c = 0.89, p = 24, nsim = 1000)

Using simulation, the calculated size (round up to the nearest 10) is 1310, more than double than the RVS-1 calculation.

We can double check our calculation is correct:

expected_cs(n = 1310, phi = 0.174, c = 0.89, p = 24, nsim = 1000)

N Mean_CS SD_CS Pr(CS<0.8) Mean_MAPE SD_MAPE

1310 0.901 0.0618 0.05 0.0335 0.0049

We note that the Monte Carlo Simulation Error with 1000 simulations at the required size to achieve the target expected CS is very small, $0.0618/\sqrt{1000}=$0.002.

# **Supplementary Tables and Figures**

#### Table S1: RvS-1 EPV versus EPV via simulation for target expected calibration slope of S=0.9, for varying prevalence and true c-statistic values

| **Prevalence** | **C-statistic** | **EPV RvS-1** | **EPV Sim** | **N RvS-1** | **N Sim** | **Inflation/Deflation required** |
| --- | --- | --- | --- | --- | --- | --- |
| 0.1 | 0.65 | 34.3 | 32.1 | 4120 | 3850 | 0.93 |
| 0.1 | 0.7 | 18.9 | 18.4 | 2260 | 2210 | 0.98 |
| 0.1 | 0.75 | 11.6 | 12.9 | 1390 | 1540 | 1.11 |
| 0.1 | 0.8 | 7.6 | 9.7 | 910 | 1160 | 1.28 |
| **0.1** | **0.85** | **5.3** | **8.0** | **640** | **960** | **1.51** |
| 0.1 | 0.9 | 3.7 | 7.3 | 440 | 880 | 1.98 |
| 0.3 | 0.65 | 44.6 | 43.5 | 1780 | 1740 | 0.98 |
| 0.3 | 0.7 | 24.6 | 28 | 980 | 1120 | 1.14 |
| 0.3 | 0.75 | 15.5 | 19.8 | 620 | 790 | 1.28 |
| 0.3 | 0.8 | 10.2 | 14.6 | 410 | 580 | 1.44 |
| **0.3** | **0.85** | **7.2** | **12.8** | **290** | **510** | **1.76** |
| 0.3 | 0.9 | 5.2 | 12.4 | 210 | 500 | 2.40 |
| 0.5 | 0.65 | 61.7 | 61.5 | 1480 | 1480 | 1.00 |
| 0.5 | 0.7 | 34.5 | 39.4 | 830 | 940 | 1.14 |
| 0.5 | 0.75 | 21.8 | 26.9 | 520 | 640 | 1.24 |
| 0.5 | 0.8 | 14.5 | 21 | 350 | 500 | 1.45 |
| **0.5** | **0.85** | **10.2** | **19.4** | **250** | **460** | **1.89** |
| 0.5 | 0.9 | 7.3 | 17.9 | 180 | 430 | 2.46 |

#### Table S2: RvS-2 EPV versus EPV via simulation for target MAPE=prevalence/10

| **Prevalence** | **C-statistic** | **EPV RvS-2** | **EPV Sim** | **N**  **RvS-2** | **N**  **Sim** | **Inflation/Deflation required** |
| --- | --- | --- | --- | --- | --- | --- |
| 0.1 | 0.65 | 51.9 | 58.6 | 6230 | 7040 | 1.13 |
| 0.1 | 0.7 | 51.9 | 56.9 | 6230 | 6820 | 1.1 |
| 0.1 | 0.75 | 51.9 | 55.4 | 6230 | 6650 | 1.07 |
| 0.1 | 0.8 | 51.9 | 51.3 | 6230 | 6160 | 0.99 |
| **0.1** | **0.85** | **51.9** | **47.2** | **6230** | **5670** | **0.91** |
| 0.1 | 0.9 | 51.9 | 41 | 6230 | 4920 | 0.79 |
| 0.3 | 0.65 | 34.9 | 44.8 | 1400 | 1790 | 1.28 |
| 0.3 | 0.7 | 34.9 | 42 | 1400 | 1680 | 1.2 |
| 0.3 | 0.75 | 34.9 | 39.2 | 1400 | 1570 | 1.12 |
| 0.3 | 0.8 | 34.9 | 36 | 1400 | 1440 | 1.03 |
| **0.3** | **0.85** | **34.9** | **31.8** | **1400** | **1270** | **0.91** |
| 0.3 | 0.9 | 34.9 | 25.6 | 1400 | 1020 | 0.73 |
| 0.5 | 0.65 | 29 | 31.9 | 700 | 760 | 1.1 |
| 0.5 | 0.7 | 29 | 30 | 700 | 720 | 1.03 |
| 0.5 | 0.75 | 29 | 28.1 | 700 | 680 | 0.97 |
| 0.5 | 0.8 | 29 | 25 | 700 | 600 | 0.86 |
| **0.5** | **0.85** | **29** | **21.9** | **700** | **520** | **0.75** |
| 0.5 | 0.9 | 29 | 18.1 | 700 | 440 | 0.62 |

#### Table S3: Type of predictors. Mean calibration slope (size calculated using either RvS-1 or simulation for target expected calibration slope of S = 0.9) and mean MAPE (size calculated using either RvS-2 or simulation for target MAPE=0.025) when the predictors are continuous and correlated or binary and unrelated. The sample size shown was obtained assuming predictors with the same distribution and strength as in the main simulation. Based on 2000 simulations.

| **Target Expected CS = 0.9** | | | |  |  |  |  |
| --- | --- | --- | --- | --- | --- | --- | --- |
|  |  |  |  | Expected CS (Mean CS over 2000 sims) | | | |
| Calc. | Size | $\phi$ | c-stat | Continuous Corr = 0 | Continuous Corr = 0.5 | Continuous Corr=0.8 | Binary Unrelated |
| RvS-1 | 4110 | 0.1 | 0.65 | 0.907 | 0.912 | 0.907 | 0.912 |
| RvS-1 | 1390 | 0.1 | 0.75 | 0.895 | 0.892 | 0.887 | 0.897 |
| RvS-1 | 640 | 0.1 | 0.85 | 0.860 | 0.858 | 0.858 | 0.863 |
| By Sim | 3760 | 0.1 | 0.65 | 0.902 | 0.902 | 0.9 | 0.907 |
| By Sim | 1540 | 0.1 | 0.75 | 0.907 | 0.907 | 0.905 | 0.907 |
| By Sim | 940 | 0.1 | 0.85 | 0.902 | 0.900 | 0.900 | 0.905 |

| **Target MAPE = 0.025** | | | |  |  |  |  |
| --- | --- | --- | --- | --- | --- | --- | --- |
|  |  |  |  | Expected MAPE (Mean MAPE over 2000 sims) | | | |
| Calc. | Size | $\phi$ | c-stat | Continuous Corr = 0 | Continuous Corr = 0.5 | Continuous Corr=0.8 | Binary Unrelated |
| RvS-2 | 1160 | 0.1 | 0.65 | 0.0249 | 0.0249 | 0.0251 | 0.0252 |
| RvS-2 | 1160 | 0.1 | 0.75 | 0.0242 | 0.0242 | 0.0242 | 0.0247 |
| RvS-2 | 1160 | 0.1 | 0.85 | 0.0225 | 0.0222 | 0.0221 | 0.0227 |
| By Sim | 1140 | 0.1 | 0.65 | 0.0251 | 0.0250 | 0.0251 | 0.0253 |
| By Sim | 1090 | 0.1 | 0.75 | 0.0246 | 0.0248 | 0.0250 | 0.0250 |
| By Sim | 960 | 0.1 | 0.85 | 0.0249 | 0.0249 | 0.0248 | 0.0250 |

#### Table S4: Time-to-event outcomes. Mean calibration slope (size calculated using RvS-1 for target expected calibration slope of S=0.9) for 12 predictor variables. Based on 2000 simulations.

| **Proportion of events** | | **Censoring proportion** | | **C-index** | | $\boldsymbol{R}_{\boldsymbol{CS}}^{\boldsymbol{2}}$ **(after censoring)** | | **n** | | **Mean CS** | |
| --- | --- | --- | --- | --- | --- | --- | --- | --- | --- | --- | --- |
| 0.1 | | 0.9 | | 0.65 | | 0.03 | | 3290 | | 0.91 | |
| 0.5 | | 0.5 | | 0.65 | | 0.14 | | 725 | | 0.9 | |
| 0.9 | | 0.1 | | 0.65 | | 0.22 | | 435 | | 0.89 | |
| 0.1 | | 0.9 | | 0.7 | | 0.06 | | 1652 | | 0.9 | |
| 0.5 | | 0.5 | | 0.7 | | 0.25 | | 377 | | 0.88 | |
| 0.9 | | 0.1 | | 0.7 | | 0.35 | | 241 | | 0.87 | |
| 0.1 | | 0.9 | | 0.75 | | 0.11 | | 913 | | 0.9 | |
| 0.5 | | 0.5 | | 0.75 | | 0.37 | | 228 | | 0.85 | |
| 0.9 | | 0.1 | | 0.75 | | 0.5 | | 151 | | 0.84 | |
| 0.1 | | 0.9 | | 0.8 | | 0.17 | | 568 | | 0.86 | |
| 0.5 | | 0.5 | | 0.8 | | 0.5 | | 149 | | 0.82 | |
| 0.9 | | 0.1 | | 0.8 | | 0.64 | | 98 | | 0.8 | |
| 0.1 | | 0.9 | | 0.85 | | 0.26 | | 360 | | 0.8 | |
| 0.5 | | 0.5 | | 0.85 | | 0.63 | | 99 | | 0.76 | |
| 0.9 | | 0.1 | | 0.85 | | 0.77 | | 63 | | 0.71 | |

#### Figure S1: Boxplots of calibration slope for different values of model strength and outcome prevalence, using the sample size calculated using the RvS-1 calibration equation with target $S=0.90$. Based on 2000 simulations.


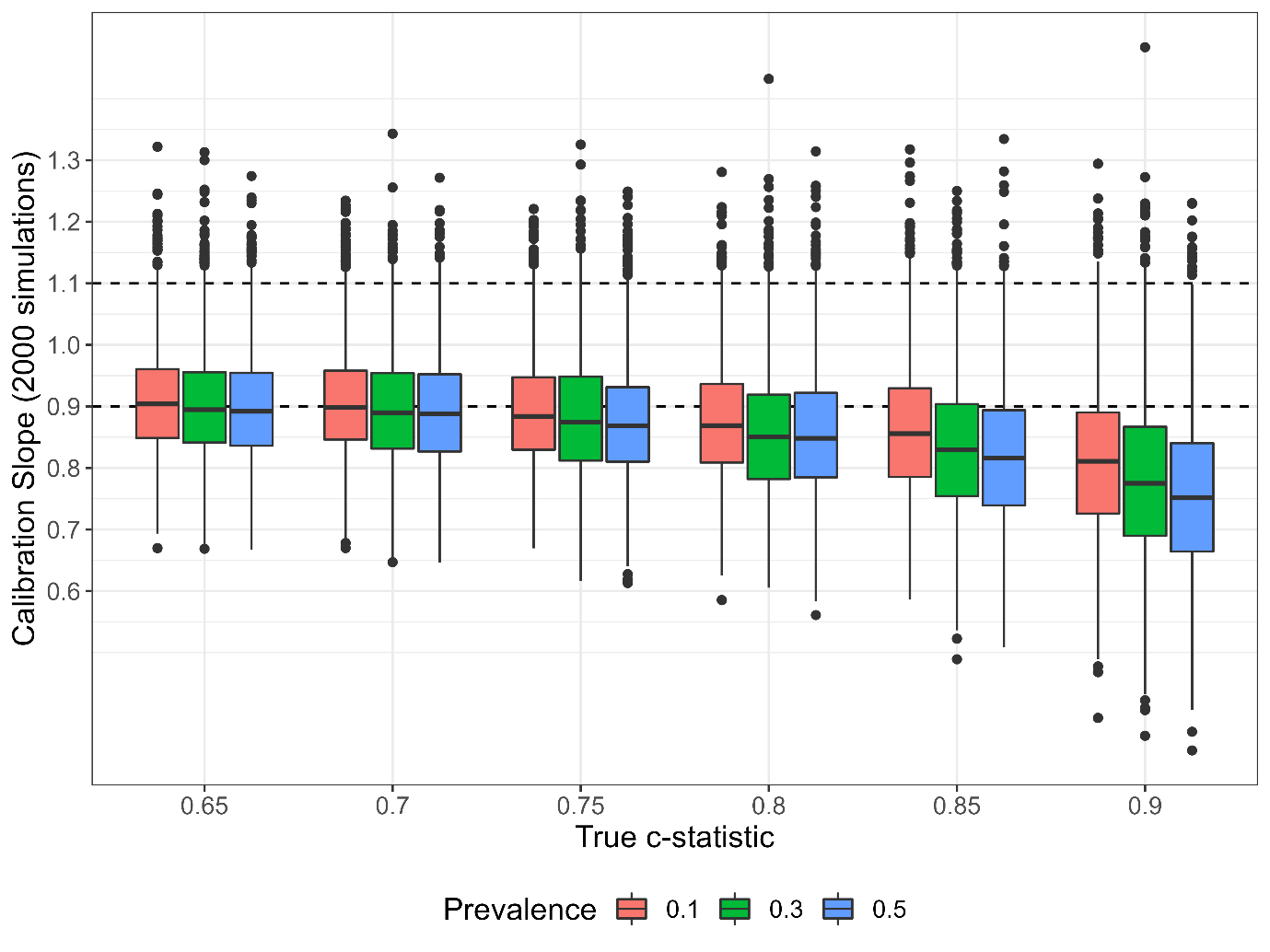


#### Figure S2: The proportion of the calculated c-statistics for estimated model within 0.02 of the true value of the c-statistic (for the true model) for different values of model strength and outcome prevalence, using: a) the sample size calculated using the RvS-1 calibration equation with target $S=0.90$ (left) and b) the actual sample size required to achieve the target expected CS (right). Based on 2000 simulations.


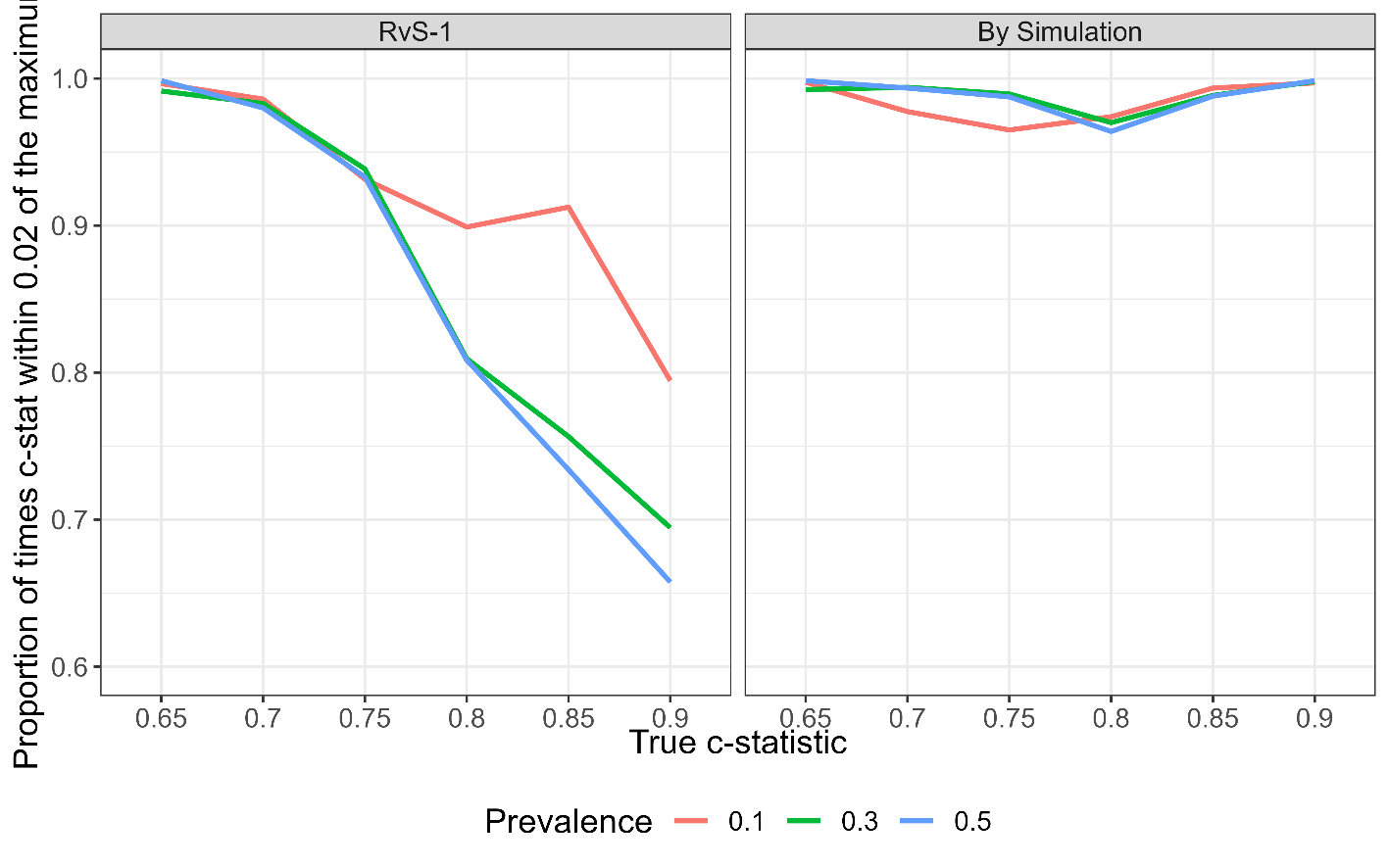


#### Figure S3: Boxplots of MAPE for different values of model strength and outcome prevalence, using the sample size calculated using the RvS-2 MAPE formula with target $MAPE m=prevalnce/10$. Based on 2000 simulations.


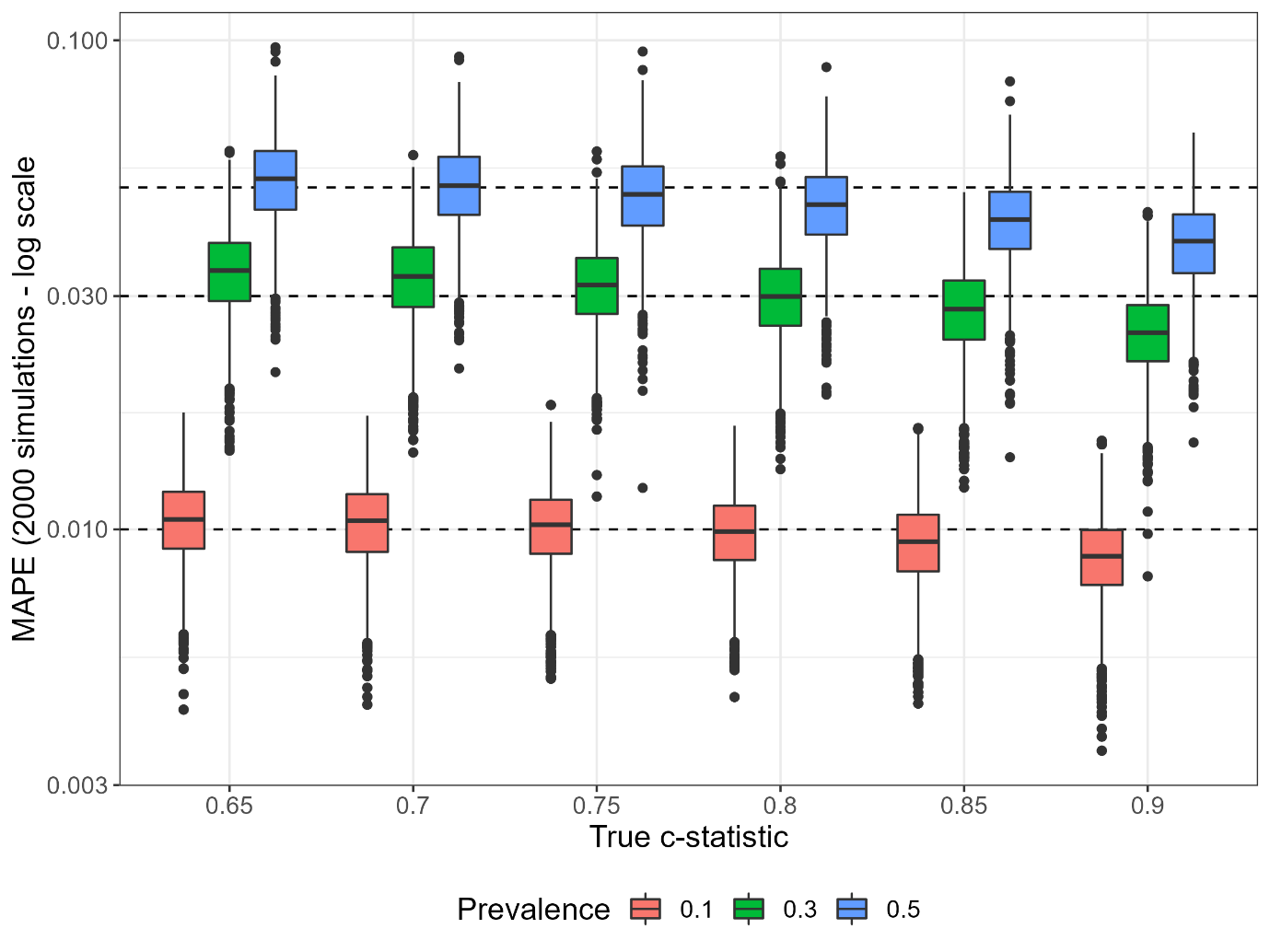


#### Figure S4: The mean calibration slope (with a 95% Confidence Interval) and the RMSD of the calibration slope while varying the number of predictor variables, at the recommended sizes (shown just above each interval) using formula RvS-1.


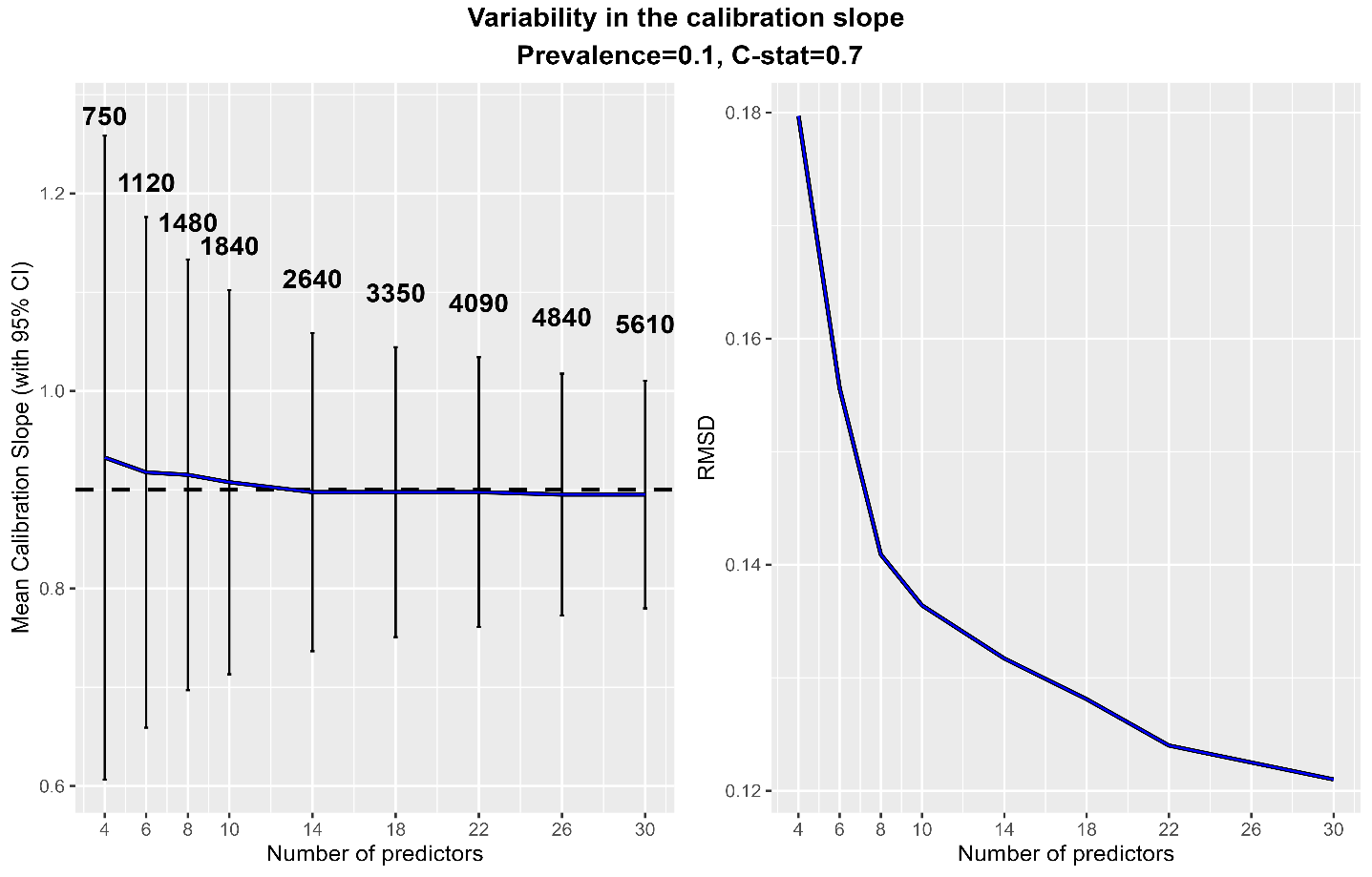

Supplement: Supplementary file 1 — Supplementary Material 1 [file 12874_2024_2268_MOESM1_ESM.docx]
