# Supplementary material for: An evaluation of sample size requirements for developing risk prediction models with binary outcomes
Source: BMC Med Res Methodol. 2024 Jul 10;24:146. doi: 10.1186/s12874-024-02268-5 (PMC11234534; doi:10.1186/s12874-024-02268-5)
Supplement: Supplementary file 2 — Supplementary Material 2 [file 12874_2024_2268_MOESM2_ESM.docx]

# R code for the paper

# 'An Evaluation of Sample Size Requirements for Developing

# Risk Prediction Models with Binary Outcomes'

# By Menelaos Pavlou

# 27/02/2024

##################################################################################

# Part A - Load necessary functions

# Scroll down to part B to run the simulation

##################################################################################

# Functions for main simulations in the paper

# 'Evaluation of sample size requirements for the development of prediction

# models for binary outcomes'

# 07/08/2023

###################################################################################

# Function to calculate the expected calibration slope and MAPE

# Inputs/Parameters

# n (numeric) : The sample size

# p (numeric) : The anticipated outcome prevalence

# c (numeric) : The anticipated c-statistic

# n.predictors (numeric) : The number of candidate predictor variables

# cor0 (numeric) : correlation between true predictors

# cor1 (numeric) : Correlation between noise predictors

# nsim (numeric) : the number of simulations

# nval (numeric) : the size of validation data

# parallel (logical) : parallel processing to speed up computations (default=TRUE)

# method (character) : the fitting method. "MLE" is the default and currently only option

# parallel (numeric) : relative strength of predictor variables (same length as n_predictors)

# beta (numeric) : the Strength of predictors (same length as n.predictors), adds up to one

# long (logical) : extract results in long format (for main simulations) or aggregated format

# Output:

# performance: the expected calibration slope and MAPE, along with standard deviations and

# and plot

expected_cs_mape_binary_corr <- function(n, p, c, beta = rep(1/n.predictors, n.predictors), n.predictors, nsim = 1000, nval = 25000, cor0=0, cor1=0, method ="MLE", parallel=TRUE, long=TRUE, r2true=FALSE){

# Find mean and variance for Normal linear predictor

set.seed(2022)

mean_var <- find_mu_sigma(p, c, tol = 0.0001)

mean <- mean_var[1]

variance <- mean_var[2]

# Find beta that corresponds to that variance

if (cor0==0 & cor1 ==0) {

beta <- beta * sqrt(mean_var[2]/sum(beta^2))

sigma <- diag(1, n.predictors)} else

{

beta <- adjust_multiplier_correlated(c=c, mean = mean, beta = beta, n.predictors = n.predictors, cor0=cor0, cor1=cor1)

beta

n.noise <- length(beta[beta==0])

n.true <- n.predictors-n.noise

# Specify correlation matrix

sigma <- matrix(0, nrow = n.predictors, ncol = n.predictors)

sigma[1:n.true, 1:n.true] <- cor0

if (n.noise>0) {

sigma[(n.true+1):n.predictors, (n.true+1):n.predictors] <- cor1}

diag(sigma) <- 1

}

if (r2true==TRUE) {

# True R2

MaxR2 <- 1-(((p^(p))*((1-p)^(1-p)))^2)

ncalc <- 1000000

x <- mvtnorm::rmvnorm(ncalc, rep(0, n.predictors), sigma = sigma )

y <- stats::rbinom( ncalc, 1, invlogit(mean + x%*%beta))

data.calc <- data.frame(y,x)

fit <- glm(y ~ ., data = data.calc, family = 'binomial')

LR <- -2 * (as.numeric(logLik(glm(y ~ 1, data = data.calc,

family = binomial(link = "logit")))) -

as.numeric(logLik(fit)))

r2 <- 1 - exp(-LR/ncalc)

n_init <- round((n.predictors)/ ((S-1)*log(1-r2/S)))

n <-n_init

# pmsampsize(type = "b", rsquared = r2, parameters =n.predictors, prevalence = p)

}

# Approximate R2

# r2 <- as.numeric(approximate_R2(c_est, p, n = 2000000)[2])

# n_init <- round((n.predictors)/ ((S-1)*log(1-r2/S)))

# n_init

xval <- mvtnorm::rmvnorm(nval, rep(0, n.predictors), sigma = sigma)

#yval <- stats::rbinom(nval, 1, invlogit(mean + xval%*%beta))

if (parallel==TRUE) {

cores <- parallel::detectCores()

cl <- parallel::makeCluster(cores[1]-2)} else

cl <- parallel::makeCluster(2)

doParallel::registerDoParallel(cl)

`%dopar%` <- foreach::`%dopar%`

`%do%` <- foreach::`%do%`

cs <- NULL

mape <- NULL

cstat_est <- NULL

R2_Nag <- NULL

i <- 0

if (method== "MLE") {

a<- foreach::foreach(i = 1:nsim, .packages=c('mvtnorm','RcppNumerical', 'ggplot2' )) %dopar% {

set.seed(i)

invlogit <- function(x) 1/(1+exp(-x))

x <- mvtnorm::rmvnorm(round(n), rep(0, n.predictors), sigma = sigma )

y <- stats::rbinom(round(n), 1, invlogit(mean + x%*%beta))

yval <- stats::rbinom(nval, 1, invlogit(mean + xval%*%beta))

p_true <- as.vector(invlogit(mean + xval%*%beta))

a <- RcppNumerical::fastLR(cbind(1,x), y)

eta_est <- cbind(1, xval) %*% as.vector(a$coef)

p_est <- as.vector(invlogit(eta_est))

fit <- RcppNumerical::fastLR(cbind(1,eta_est), yval, start = c(0,0.9) )

cs[i] <- fit$coef[2]

mape[i] <- mean(abs(p_true-p_est))

#NEW

# cstat_est[i] <- quickcstat(yval, p_est)

cstat_est[i] <- pROC::roc(as.vector(yval), as.vector(eta_est), quiet=TRUE)$auc

c(cs[i],mape[i], cstat_est[i])

}

} else if (method == "LSF")

{

bootsf<-function(data,n=100){

#first column outcome

cal<-NULL

for (j in 1:n){

bs <- sample(nrow(data), replace=T)

databs=data[bs,]

xvarsbs=databs[,-1];ybs<-databs[,1]

fitbs <- speedglm::speedglm(ybs~xvarsbs, family=binomial())

eta_est <- as.matrix(cbind(1,data[,-1]))%*%coef(fitbs)

fitcal <- speedglm::speedglm(data[,1]~eta_est, family=binomial())

cal[j] <- as.vector(stats::coef(fitcal)[2])

}

return(stats::median(cal,na.rm=TRUE))

}

a<- foreach::foreach(i = 1:nsim, .packages=c('mvtnorm','RcppNumerical', 'ggplot2', 'speedglm' )) %dopar% {

set.seed(i)

invlogit <- function(x) 1/(1+exp(-x))

x <- mvtnorm::rmvnorm(round(n), rep(0, n.predictors), sigma = sigma )

y <- stats::rbinom(round(n), 1, invlogit(mean + x%*%beta))

yval <- stats::rbinom(nval, 1, invlogit(mean + xval%*%beta))

p_true <- as.vector(invlogit(mean + xval%*%beta))

a <- RcppNumerical::fastLR(cbind(1,x), y)

datasf <- cbind(y, x)

sf <- bootsf(datasf, 100)

betasf <- c(1,rep(sf,n.predictors))*a$coef

off <- speedglm::speedglm(y~1,offset=cbind(1,x)%*%betasf,family=binomial())

betasf[1] <- betasf[1]+stats::coef(off)

eta_est <- cbind(1, xval)%*%betasf

p_est <- as.vector(invlogit(eta_est))

fit <- RcppNumerical::fastLR(cbind(1,eta_est), yval )

cs[i] <- fit$coef[2]

mape[i] <- mean(abs(p_true-p_est))

#NEW

cstat_est[i] <-quickcstat(yval, p_est)

c(cs[i],mape[i], cstat_est[i])

}

}

parallel::stopCluster(cl)

b <- matrix(unlist(a), byrow=TRUE, nrow=nsim)

cs <- b[,1]

mape <- b[,2]

#NEW

cstat_est <- b[,3]

df <- data.frame(cs)

df <- stats::na.omit(df)

cs_plot <- ggplot2:: ggplot(df, ggplot2::aes(x = cs), size=15) +

ggplot2::geom_density() + ggplot2::ggtitle(paste("Mean Calibration Slope = ",round(mean(cs,na.rm=TRUE),3))) +

ggplot2::geom_vline( ggplot2::aes(xintercept = mean(cs, na.rm = TRUE)), color="blue", linetype ="dashed", size = 1) +

ggplot2::ylab("Density") + ggplot2::theme(text = ggplot2::element_text(size = 14)) +

ggplot2::xlab("Calibration Slope") + ggplot2::theme_bw()+ ggplot2::theme(legend.position="bottom") + theme(text = element_text(size = 15))

if ( abs(mean(cs, na.rm=TRUE)- 0.9) > 0.005) cs_plot <- cs_plot + ggplot2::geom_vline( ggplot2::aes(xintercept = 0.9), color="red", linetype ="dashed", size = 1)

df <- data.frame(mape)

df <- stats::na.omit(df)

mape_plot <- ggplot2::ggplot(df, ggplot2::aes(x = mape), size=15) +

ggplot2::geom_density() + ggplot2::ggtitle(paste("Mean MAPE = ", round(mean(mape,na.rm=TRUE),3), sep = "")) +

ggplot2::geom_vline( ggplot2::aes(xintercept=mean(mape, na.rm = TRUE)), color="blue", linetype = "dashed", size=1) + ggplot2::ylab("Density") +

ggplot2::theme(text = ggplot2::element_text(size = 14)) +ggplot2::xlab("MAPE") +

ggplot2::theme_bw()+ ggplot2::theme(legend.position="bottom") + theme(text = element_text(size = 15))

figure <- ggpubr::ggarrange(cs_plot, mape_plot,

ncol = 2, nrow = 1, common.legend = TRUE, legend="bottom")

cs_mape_plot <- ggpubr::annotate_figure(figure,

top = ggpubr::text_grob(paste("Distribution of the Calibration Slope and MAPE\n","N=", n, ", Prevalence=", p, ", C-stat=",c,sep=""),

color = "black", face = "bold", size = 14)) + ggplot2::xlab("MAPE")

print(cs_mape_plot)

# cs_mape_plot

# ggsave("plots development//Figure_6.png", width=12, height=6, dpi=300)

#

set.seed(2022)

xval <- mvtnorm::rmvnorm(2000000, rep(0,n.predictors), sigma = sigma)

yval <- stats::rbinom(2000000, 1, invlogit(mean + xval %*% beta))

prev <- mean(yval)

cstat <- quickcstat(yval, invlogit(mean + xval %*% beta))

# cstat <- pROC::roc(as.vector(yval), invlogit(mean + xval %*% beta), quiet=TRUE)$auc

# Short format for presentation

df <- data.frame(n, ceiling(mean(cs, na.rm = TRUE)/0.0025) * 0.0025,

round(sqrt(stats::var(cs,na.rm = TRUE)), 4),

round(sqrt( mean( ((cs-1)^2), na.rm=TRUE) ), 4),

round(mean(ifelse( (cs < 0.8), 1, 0),na.rm=TRUE), 2),

round(mean(mape, na.rm = TRUE),4),

round(sqrt(stats::var(mape,na.rm = TRUE)), 4),

round(prev, 4),

round(cstat, 4 ),

n.predictors)

names(df) <- c("N", "Mean_CS", "SD_CS", "RMSD_CS", "Pr(CS<0.8)", "Mean_MAPE", "SD_MAPE", "Prev.", "C-Stat.", " # Predictors")

performance <- df[,-3]

performance1 <- df

# Long format for simulations

performance2 <- data.frame(n, n.predictors, round(cstat,3), round(prev, 3), cs, mape, cstat_est)

names(performance2) = c("n", "npred", "cstat","prev","cs_est","mape_est","cstat_est")

performance2

if (long==TRUE) performance <- performance2 else performance <- performance1

performance

}

# Example

# expected_cs_mape_binary_corr(n = 530, p = 0.2, c = 0.85, n.predictors = 10, long=FALSE)

#################################################################################################################

# Function to calculate Sample size required to develop a risk prediction model based on the

# CALIBRATION SLOPE

# Inputs

# S (numeric) : the target expected shrinkage (targert calibration slope)

# p (numeric) : the anticipated outcome prevalence

# c (numeric) : the anticipated C-statistic

# n.predictors : the number of candidate predictor variables

# nsim (numeric) : the number of simulations (>=500, default value 1000 to ensure small simulation error)

# nval (numeric) : Size of validation data (at least 25000)

# parallel(logical) : parallel processing to speed up computations (default=TRUE)

# Outputs: the required sample size to achieve required calibration slope

# (Actual by Simulation and RvS-1 formula for comparison)

samplesizedev_binary_s_corr <- function(S, p, c, n.predictors, beta=rep(1/n.predictors, n.predictors), nval = 25000, nsim = 1000, parallel = TRUE, cor0=0.1, cor1=0.05, r2true=TRUE, acc=0.0025){

set.seed(2024)

mean_var <- find_mu_sigma(p, c, tol = 0.0001)

mean_eta <- mean_var[1]

variance_eta <- mean_var[2]

# Find beta that corresponds to that variance

if (cor0==0 & cor1 ==0) {

betan <- beta * sqrt(mean_var[2]/sum(beta^2))

sigma <- diag(1, n.predictors)} else

{

betan <- adjust_multiplier_correlated(c=c, mean = mean_eta, beta = beta, n.predictors = n.predictors, cor0=cor0, cor1=cor1)

n.noise <- length(beta[beta==0])

n.true <- n.predictors-n.noise

# Specify correlation matrix

sigma <- matrix(0, nrow = n.predictors, ncol = n.predictors)

sigma[1:n.true, 1:n.true] <- cor0

if (n.noise>0) {

sigma[(n.true+1):n.predictors, (n.true+1):n.predictors] <- cor1}

diag(sigma) <- 1

}

if (r2true==TRUE) {

# True R2

MaxR2 <- 1-(((p^(p))*((1-p)^(1-p)))^2)

ncalc <- 2000000

x <- mvtnorm::rmvnorm(ncalc, rep(0, n.predictors), sigma = sigma )

y <- stats::rbinom( ncalc, 1, invlogit(mean_eta + x%*%betan))

data.calc <- data.frame(y,x)

fit <- glm(y ~ ., data = data.calc, family = 'binomial')

LR <- -2 * (as.numeric(logLik(glm(y ~ 1, data = data.calc,

family = binomial(link = "logit")))) -

as.numeric(logLik(fit)))

r2 <- 1 - exp(-LR/ncalc)

n_init <- round((n.predictors)/ ((S-1)*log(1-r2/S)))

n_init} else {

# Approximation R2

r2 <- as.numeric(approximate_R2(c, p, n = 2000000)[2])

n_init <- round((n.predictors)/ ((S-1)*log(1-r2/S)))

n_init

}

if (c<=0.7) {inflation_f <- 1.3 ; min.opt <- n_init*0.65}

if (c>0.7 & c<=0.8) {inflation_f <- 1.6 ; min.opt <- n_init*0.95}

if (c>0.8 & c<=0.85) {inflation_f <- 2.1 ; min.opt <- n_init*1.2}

if (c>0.85 & c<=0.9) {inflation_f <- 2.8 ; min.opt <- n_init*1.4}

max.opt <- inflation_f*n_init

if (n_init > 2100) tol = ceiling(round(n_init/400)/5) * 5 else tol = ceiling(round(n_init/200)/5)

print("Optimisation Starting ~ 1 min left...")

s_est <- function(n, nsim=nsim){

s <- expected_s_n_binary_corr(n, S = S, mean_eta = mean_eta, variance_eta = variance_eta, beta = betan, p = p, c = c, n.predictors = n.predictors, nval = nval, nsim = nsim, parallel=parallel, cor0=cor0, cor1=cor1, acc=acc)

s[1] - S

}

n <- bisection(s_est, min.opt, max.opt, tol = tol, nsim = nsim, acc = acc)

if (n > 2100) tol = ceiling(round(n/400)/5) * 5 else tol = ceiling(round(n/200)/5)

n <- ceiling(n/5)*5

size <- NULL

size$rvs1 <- as.vector(round(n_init))

size$actual <- as.vector(round(n))

size$correct_to_nearest <- as.vector(tol)

size

}

#######################################################################################################

# Function to calculate the expected calibration slope for a given sample size

# Feed into the function for sample size calculations based on shrinkage (calibration slope)

# Inputs same as above

# Outputs: mean calibration slope

expected_s_n_binary_corr <- function(n, S, mean_eta, variance_eta, p, c, n.predictors, beta, nsim = 1000, nval = 25000, parallel = TRUE, cor0, cor1, acc){

set.seed(2023)

# Specify correlation matrix

if (cor0==0 & cor1 ==0) {

sigma <- diag(1, n.predictors)} else

{

n.noise <- length(beta[beta==0])

n.true <- n.predictors-n.noise

sigma <- matrix(0, nrow = n.predictors, ncol = n.predictors)

sigma[1:n.true, 1:n.true] <- cor0

if (n.noise>0) {

sigma[(n.true+1):n.predictors, (n.true+1):n.predictors] <- cor1}

diag(sigma) <- 1

}

xval <- mvtnorm::rmvnorm(nval, rep(0, n.predictors), sigma = sigma)

#yval <- stats::rbinom(nval, 1, invlogit(mean_eta + xval%*%beta))

if (parallel==TRUE) {

cores <- parallel::detectCores()

cl <- parallel::makeCluster(cores[1]-2)} else

cl <- parallel::makeCluster(2)

doParallel::registerDoParallel(cl)

`%dopar%` <- foreach::`%dopar%`

`%do%` <- foreach::`%do%`

cs <- NULL

i <- 0

a <- foreach::foreach(i=1:nsim, .packages=c('mvtnorm','RcppNumerical', 'ggplot2' )) %dopar% {

set.seed(i)

invlogit<-function(x) 1/(1+exp(-x))

x <- mvtnorm::rmvnorm(round(n), rep(0, n.predictors), sigma = sigma )

y <- stats::rbinom(round(n), 1, invlogit(mean_eta + x%*%beta))

yval <- stats::rbinom(nval, 1, invlogit(mean_eta + xval%*%beta))

#a <- fastglm(cbind(1,x), y, family=binomial())

a <- RcppNumerical::fastLR(cbind(1,x), y)

eta_est <- cbind(1, xval) %*% as.vector(a$coef)

#fit <- fastglm(cbind(1,eta_est), yval, family=binomial())

fit <- RcppNumerical::fastLR(cbind(1,eta_est), yval)

cs[i] <- fit$coef[2]

cs[i]

}

parallel::stopCluster(cl)

cs <- unlist(a)

#graphics::hist(cs, main = paste("CS=",round(mean(cs,na.rm=TRUE)/0.0025)*0.0025, "N=",n))

yval <- stats::rbinom(nval, 1, invlogit(mean_eta + xval%*%beta))

df <- data.frame(cs)

df <- stats::na.omit(df)

cs_plot <- ggplot2:: ggplot(df, ggplot2::aes(x = cs), size=12) +

ggplot2::geom_density() + ggplot2::ggtitle(paste("N = ", round(n), "p = ", mean(yval), ", Expected CS = ", round(mean(cs,na.rm=TRUE)/acc)*acc, ", SD(CS) = ", round(sqrt(stats::var(cs,na.rm=TRUE)),3))) +

ggplot2::geom_vline( ggplot2::aes(xintercept = mean(cs, na.rm = TRUE)), color="blue", linetype ="dashed", size = 1) +

ggplot2::ylab("Density") + ggplot2::theme(text = ggplot2::element_text(size = 13)) +

ggplot2::xlab("Calibration Slope")

if ( abs(mean(cs, na.rm=TRUE)- 0.9) > 0.005) cs_plot <- cs_plot + ggplot2::geom_vline( ggplot2::aes(xintercept = 0.9), color="red", linetype ="dashed", size = 1)

print(cs_plot)

c(round(mean(cs,na.rm=TRUE)/acc)*acc, sqrt(stats::var(cs)/nsim))

# c(round(mean(cs,na.rm=TRUE)/0.002)*0.002, sqrt(stats::var(cs)/nsim))

}

#################################################################################################################

# Function to calculated Sample size required to develop a risk prediction model based on MAPE

# Inputs

# MAPE (numeric) : The target expected MAPE

# p (numeric) : The anticipated outcome prevalence

# c (numeric) : The anticipated C-statistic

# n.predictors :(numeric) The number of candidate predictor variables

# nsim (numeric) : The number of simulations (at least 500, default value 1000 to ensure small simulation error)

# nval (numeric) : Size of validation data (at least 10000 )

# parallel(logical): parallel processing to speed up computations (default=TRUE)

# Outputs: The required sample size to achieve required MAPE (Actual by Simulation and RvS-2 for comparison)

samplesizedev_binary_mape_corr <- function(MAPE, p, c, n.predictors, beta, nval = 25000, nsim = 1000, parallel = TRUE, cor0=0, cor1=0){

set.seed(2022)

mean_var <- find_mu_sigma(p,c)

mean_eta <- mean_var[1]

variance_eta <- mean_var[2]

# Find beta that corresponds to that variance

if (cor0==0 & cor1 ==0) {

betan <- beta * sqrt(mean_var[2]/sum(beta^2))

sigma <- diag(1, n.predictors)} else

{

betan <- adjust_multiplier_correlated(c=c, mean = mean_eta, beta = beta, n.predictors = n.predictors, cor0=cor0, cor1=cor1)

n.noise <- length(beta[beta==0])

n.true <- n.predictors-n.noise

# Specify correlation matrix

sigma <- matrix(0, nrow = n.predictors, ncol = n.predictors)

sigma[1:n.true, 1:n.true] <- cor0

if (n.noise>0) {

sigma[(n.true+1):n.predictors, (n.true+1):n.predictors] <- cor1}

diag(sigma) <- 1

}

n_init <- exp((-0.508 + 0.259 * log(p) + 0.504 * log(n.predictors) - log(MAPE))/0.544) ;

min.opt = round(n_init*0.5)

max.opt = round(n_init*1.5)

tol = ceiling(round(n_init/200)/5) * 5

print("Optimisation Starting ~ 1 min left...")

mape_est <- function(n, nsim=nsim){

mape <- expected_mape_n_binary_corr(n, MAPE = MAPE, mean_eta = mean_eta, variance_eta = variance_eta, beta=betan, p = p, c = c, n.predictors = n.predictors, nval = nval, nsim = nsim, parallel = parallel, cor0=cor0)

MAPE-mape[1]

}

n <- bisection_mape(mape_est, MAPE=MAPE, min.opt, max.opt, tol = tol, nsim = nsim)

tol = ceiling(round(n/200)/5) * 5

n <- ceiling(n/tol)*tol

size <- NULL

size$rvs2 <- as.vector(round(n_init))

size$actual <- as.vector(round(n))

size

}

#######################################################################################################

# Function to calculate the expected calibration slope for a given sample size

# Feed into the function for sample size calculations based on MAPE

# Inputs same as above

# Outputs: mean MAPE

expected_mape_n_binary_corr <- function(n, MAPE, mean_eta, variance_eta, p, c, beta, n.predictors, nsim = 1000, nval = 25000, parallel = TRUE, cor0=0, cor1=0){

set.seed(2022)

# Find beta that corresponds to that variance

if (cor0 == 0 & cor1 == 0)

sigma <- diag(1, n.predictors) else {

n.noise <- length(beta[beta==0])

n.true <- n.predictors-n.noise

sigma <- matrix(0, nrow = n.predictors, ncol = n.predictors)

sigma[1:n.true, 1:n.true] <- cor0

if (n.noise>0) {

sigma[(n.true+1):n.predictors, (n.true+1):n.predictors] <- cor1}

diag(sigma) <- 1

}

xval <- mvtnorm::rmvnorm(nval, rep(0, n.predictors), sigma = sigma)

if (parallel==TRUE) {

cores <- parallel::detectCores()

cl <- parallel::makeCluster(cores[1]-2)} else

cl <- parallel::makeCluster(2)

doParallel::registerDoParallel(cl)

`%dopar%` <- foreach::`%dopar%`

`%do%` <- foreach::`%do%`

mape <- NULL

i <- 0

a <- foreach::foreach(i=1:nsim, .packages=c('mvtnorm','RcppNumerical', 'ggplot2' )) %dopar% {

set.seed(i)

invlogit<-function(x) 1/(1+exp(-x))

x <- mvtnorm::rmvnorm(round(n), rep(0, n.predictors), sigma = sigma )

y <- stats::rbinom(round(n), 1, invlogit(mean_eta + x%*%beta))

yval <- stats::rbinom(nval, 1, invlogit(mean_eta + xval%*%beta))

p_true <- as.vector(invlogit(mean_eta + xval%*%beta))

#a <- fastglm(cbind(1,x), y, family=binomial())

a <- RcppNumerical::fastLR(cbind(1,x), y)

eta_est <- cbind(1, xval) %*% as.vector(a$coef)

p_est <- as.vector(invlogit(eta_est))

#fit <- fastglm(cbind(1,eta_est), yval, family=binomial())

fit <- RcppNumerical::fastLR(cbind(1,eta_est), yval)

mape[i] <- mean(abs(p_true-p_est))

mape[i]

}

parallel::stopCluster(cl)

mape <- unlist(a)

#graphics::hist(cs, main = paste("CS=",round(mean(cs,na.rm=TRUE)/0.0025)*0.0025, "N=",n))

df <- data.frame(mape)

df <- stats::na.omit(df)

mape_plot <- ggplot2:: ggplot(df, ggplot2::aes(x = mape), size=12) +

ggplot2::geom_density() + ggplot2::ggtitle(paste("N = ", n, ", Expected MAPE = ", round(mean(mape,na.rm=TRUE)/0.0001)*0.0001, ", SD(MAPE) = ", round(sqrt(stats::var(mape)),4))) +

ggplot2::geom_vline( ggplot2::aes(xintercept = mean(mape, na.rm = TRUE)), color="blue", linetype ="dashed", size = 1) +

ggplot2::ylab("Density") + ggplot2::theme(text = ggplot2::element_text(size = 13)) +

ggplot2::xlab("MAPE")

print(mape_plot)

c(round(mean(mape,na.rm=TRUE)/0.0001)*0.0001, sqrt(var(mape)/nsim))

}

#######################################################################################################

# Bisection method for sample size calculation (shrinkage)

# a, b: starting values

# f: function evaluated

bisection <- function(f, a, b, iter = 15, tol = ceiling(round(a/200)/5) * 5, nsim = 1000, acc = 0.0025) {

# If the signs of the function at the evaluated points, a and b, stop the function and return message.

if (a > 2100) tol = ceiling(round(a/400)/5) * 5 else tol = ceiling(round(a/200)/5)

#tol = ceiling(round(a/200)/5) * 5

nsim1 <- nsim

if (nsim>=1000) divide <- 2 else divide <- 1

nsim <- round(nsim/divide)

fa <- f(a, nsim = nsim)

# print(fa)

fb <- f(b, nsim = nsim)

# print(fb)

if (!(fa < 0) && (fb > 0)) {

stop('signs of f(a) and f(b) differ')

} else if ((fa > 0) && (fb < 0)) {

stop('signs of f(a) and f(b) differ')

}

for (k in 1:iter) {

if (k <= divide ) nsim <- nsim1/divide*k

# a <- round(a/tol)*tol

# b <- round(b/tol)*tol

c <- round ((a + b) / 2) # Calculate midpoint

fc <- f(c, nsim = nsim)

# print(fc)

# print(c(k, a, b, c, fa, fb, fc ))

#print(c(k))

# If the function equals 0 at the midpoint or the midpoint is below the desired tolerance, stop the

# function and return the root.

if ( ((abs(fc) <= acc) || ((b - a) / 2) < tol) & (k >=1 )) {

#if ( abs(fc) <= 0.0025 & (k >=2 )) {

return(c)

}

# If another iteration is required,

# check the signs of the function at the points c and a and reassign

# a or b accordingly as the midpoint to be used in the next iteration.

ifelse(sign(fc) == sign(fa),

a <- c,

b <- c)

ifelse(sign(fc) == sign(fa),

fa <- fc,

fb <- fc)

}

# If the max number of iterations is reached and no root has been found,

# return message and end function.

print('Too many iterations')

}

#############################################################################

# Bisection method for sample size calculation (MAPE)

# a, b: starting values

# f: funciton evaluated

bisection_mape <- function(f, a, b, MAPE = 0.0001, iter = 10, tol = ceiling(round(a/200)/5) * 5, nsim = 1000) {

# If the signs of the function at the evaluated points, a and b, stop the function and return message.

tol = ceiling(round(a/200)/5) * 5

nsim1 <- nsim

if (nsim>=1000) divide <- 2 else divide <- 1

nsim <- round(nsim/divide)

fa <- f(a, nsim = nsim)

fb <- f(b, nsim = nsim)

if (!(fa < 0) && (fb > 0)) {

stop('signs of f(a) and f(b) differ')

} else if ((fa > 0) && (fb < 0)) {

stop('signs of f(a) and f(b) differ')

}

for (k in 1:iter) {

if (k <= divide ) nsim <- nsim1/divide*k

# a <- round(a/tol)*tol

# b <- round(b/tol)*tol

c <- (a + b) / 2 # Calculate midpoint

fc <- f(c, nsim = nsim)

#print(c(k, a, b, c, fa, fb, fc ))

#print(c(k))

# If the function equals 0 at the midpoint or the midpoint is below the desired tolerance, stop the

# function and return the root.

if ( ((abs(fc) <= MAPE/200) || ((b - a) / 2) < tol) & (k >=2 )) {

# if ( abs(fc) <= MAPE/200 & (k >=2 )) {

return(c)

}

# If another iteration is required,

# check the signs of the function at the points c and a and reassign

# a or b accordingly as the midpoint to be used in the next iteration.

ifelse(sign(fc) == sign(fa),

a <- c,

b <- c)

ifelse(sign(fc) == sign(fa),

fa <- fc,

fb <- fc)

}

# If the max number of iterations is reached and no root has been found,

# return message and end function.

print('Too many iterations')

}

########################################################################################

# Find mean and variance of linear predictor for given prevalence

# and C-statistic (using numerical integration)

# Part of functions calculating expected shrinkage and MAPE

find_mu_sigma <- function(target.prev, target.c, min.opt = c(-10,0), max.opt = c(0.02,5), tol = 0.00001){

pcfun <- function(x){

#target.prev = 0.2; target.c=0.7; min.opt = c(-7,0.5); max.opt = c(0,14)

mean <- x[1]

variance <- x[2]

f1 = function(x) {

stats::integrate(function(y) {stats::dnorm(x, mean = mean, sd = sqrt(variance)) * stats::dnorm(y, mean = mean, sd = sqrt(variance)) * (1 + exp(-x)) ^ (-1) * (1 + exp(y)) ^ (-1) },

-Inf, x)$value

}

num = stats::integrate(Vectorize(f1), -Inf, Inf)$value

f2 = function(x) {

stats::integrate(function(y) {stats::dnorm(x, mean = mean, sd = sqrt(variance)) * stats::dnorm(y, mean = mean, sd = sqrt(variance)) * (1 + exp(-x)) ^ (-1) * (1 + exp(y)) ^ (-1) },

-Inf, Inf)$value

}

denom <- stats::integrate(Vectorize(f2), -Inf, Inf)$value

f3 <- function(x) stats::dnorm(x, mean=mean, sd = sqrt(variance)) * (1 + exp(-x)) ^ (-1)

c <- num/denom

prev <- stats::integrate(f3, - Inf, Inf, subdivisions = 1000L)$value

abs( c - target.c)^2 + abs(prev - target.prev )^2

}

if (target.c>0.65) {

out <- stats::optim(par=c(-2.65,0.1), pcfun, c(min.opt, max.opt, tol = tol))$par} else

{ sigma_c <- sqrt(2) * stats::qnorm(target.c)

mu <- 0.5 * (2 * target.prev - 1) * (sigma_c^2) + log(target.prev / (1 - target.prev))

sigma <- sqrt((sigma_c^2) * (1 + target.prev * (1 - target.prev) * (sigma_c^2)))

out <- c(mu, sigma^2)

}

N <- 2000000

lp <- stats::rnorm(N, mean = out[1], sd = sqrt(out[2]))

p <- (1 + exp(-lp)) ^ (-1)

y <- stats::rbinom(N, 1, prob = p)

prev <- mean(y)

c <- quickcstat(y, lp)

c(out[1], out[2], prev, c)

}

# Check

# round(find_mu_sigma(0.1, 0.65, tol=0.00001),4)

########################################################################################

# Riey's function (Statistics in Medicine) to approximate R2 from AUC and prevalence)

approximate_R2 <- function(auc, prev, n = 1000000, seed=1){

set.seed(seed)

# define mu as a function of the C-statistic

mu <- sqrt(2) * stats::qnorm(auc)

# sigmain <- sqrt(2)*qnorm(auc)

# mu<-0.5*(2*prev-1)*(sigmain^2)+log(prev/(1-prev))

# simulate large sample linear prediction based on two normals

# for non-eventsN(0, 1), events and N(mu, 1)

LP <- c(stats::rnorm(prev*n, mean=0, sd=1), stats::rnorm((1-prev)*n, mean=mu, sd=1))

y <- c(rep(0, prev*n), rep(1, (1-prev)*n))

# Fit a logistic regression with LP as covariate;

# this is essentially a calibration model, and the intercept and

# slope estimate will ensure the outcome proportion is accounted

# for, without changing C-statistic

fit <- rms::lrm(y~LP)

max_R2 <- function(prev){

1-(prev^prev*(1-prev)^(1-prev))^2

}

return(list(R2.nagelkerke = as.numeric(fit$stats['R2']),

R2.coxsnell = as.numeric(fit$stats['R2']) * max_R2(prev)))

}

########################################################################################

# Approximation of the C-statistic (large n)

# Part of various functions above

quickcstat <- function(y, pred, seed=1){

#set.seed(seed)

casepred=pred[y == 1]

conpred=pred[y == 0]

if (length(conpred)>length(casepred)){

conpred=conpred[sample(length(conpred),length(casepred),replace=FALSE)]

auc.true=sum(casepred>conpred)/length(casepred)} else

{

casepred=casepred[sample(length(casepred),length(conpred),replace=FALSE)]

auc.true=sum(casepred>conpred)/length(conpred)

}

return(auc.true)

}

########################################################################################

# Find coefficients for desired c-statistic and prevalence (correlated predictors)

# Part of functions calculating expected shrinkage and MAPE

adjust_multiplier_correlated <- function(c, mean, beta, n.predictors, min.opt = variance/3, max.opt = variance*3, tol=0.01, cor0, cor1){

N <- 3000000

n.noise <- length(beta[beta==0])

n.true <- n.predictors - n.noise

# mean_var <- find_mu_sigma(p, c, tol=0.001)

# beta_new <- find_multiplier(sqrt(variance), beta=beta, n.true=n.true, n.noise=n.noise)

# Specify correlation matrix

sigma <- matrix(0, nrow = n.predictors, ncol = n.predictors)

sigma[1:n.true, 1:n.true] <- cor0

if (n.noise>0) {

sigma[(n.true+1):n.predictors, (n.true+1):n.predictors] <- cor1}

diag(sigma) <- 1

x <- mvtnorm::rmvnorm(round(N), rep(0,nrow(sigma)), sigma = sigma )

cfun <- function(adjust){

eta <- mean + x %*% as.matrix(beta) * adjust

y <- stats::rbinom(N, 1, invlogit(eta))

cest <- quickcstat(y,eta)

abs(cest- c)

}

adjust <- optimize(cfun, c(0, 7, tol = 0.001))$minimum

adjust

beta_new <- beta*adjust

beta_new

# Check beta's are ok

#eta <- mean + x%*%as.matrix(beta_new)

#y <- rbinom(N, 1, invlogit(eta))

#mean(y)

#quickcstat(y, eta)

}

########################################################################################

# Find coefficients for desired c-statistic and prevalence (correlated predictors)

# Needed for the simulation results in the appendix

adjust_multiplier_binary <- function(c, p, beta = c(-0.5,-0.3,0.3,0.15,0.15), type="binary", n.true, n.noise, N=500000){

set.seed(1)

N <- 500000

beta = c(-0.5,-0.3,0.3,0.15,0.15)

beta <- c(beta,rep(0.1,n.true-5), rep(0,n.noise))

x <- rmvbin(N, margprob=c(0.7, 0.7, 0.3, 0.4, 0.5, rep(0.5,n.true-5), rep(0.2,n.noise)))

cfun <- function(b0_adjust){

b0 <- b0_adjust[1]

adjust <- b0_adjust[2]

eta <- cbind(1,x)%*%c(b0, beta*adjust)

y <- rbinom(N, 1, invlogit(eta))

cest <- pROC::roc(as.vector(y), as.vector(eta), quiet=TRUE)$auc

prev <- mean(y)

(abs(cest - c) + abs(prev-p))

}

run <- round(optim(par=c(-2.65,1), cfun, c(min.opt=c(-7,0.5), max.opt=c(6,13), tol = 0.0005))$par, 6)

b0 <- run[1]

adjust <- run[2]

# # # # #check

# set.seed(1)

# beta <- c(-0.5,-0.3,0.3,0.15,0.15)

# beta <- c(beta,rep(0.1,n.true-5), rep(0,n.noise))

# beta <- c(b0, beta*adjust)

# x2 <- rmvbin(N, margprob=c(0.7, 0.7, 0.3, 0.4, 0.5, rep(0.5,n.true-5), rep(0.2,n.noise)))

# eta2 <- cbind(1,x2)%*%beta

# mean(eta2)

# y2 <- rbinom(N, 1, invlogit(eta2))

# p_true <- invlogit(eta2)

# p_est <- mean(y2)

# c_est <- pROC::roc(as.vector(y2), as.vector(eta2), quiet=TRUE)$auc

# c_est; p_est

c(b0, beta*adjust)

}

invlogit <- function(x) 1/(1+exp(-x))

########################################################################################

# Function to calculate the expected calibration slope and MAPE (Binary predictors)

# Inputs/Parameters

expected_cs_binary_binarypred <- function(n, S, p, c, beta = c(-0.5,-0.3,0.3,0.15,0.15) , n.true=5, n.noise=7, r2=0, nsim=500, nval=50000, r2true = FALSE){

beta = c(-0.5,-0.3,0.3,0.15,0.15)

n.predictors <- n.true + n.noise

set.seed(2022)

beta <- adjust_multiplier_binary(c, p, beta = beta, n.true = n.true, n.noise = n.noise)

cs <- NULL

mape <- NULL

# xval1 <- rmvbin(1000000, margprob=c(0.7, 0.7, 0.3, 0.4, 0.5, rep(0.2,n.noise)))

# yval <- rbinom(1000000, 1, invlogit((cbind(1,xval1)%*%beta)))

# p_true <- invlogit(as.matrix(cbind(1,xval1))%*%beta)

# p_est <- mean(yval)

# c_est <- pROC::roc(as.vector(yval), as.vector(p_true), quiet=TRUE)$auc

# p_est

# c_est

if (r2true==TRUE) {

MaxR2 <- 1-(((p^(p))*((1-p)^(1-p)))^2)

ncalc <- 1000000

x <- rmvbin(round(ncalc), margprob=c(0.7, 0.7, 0.3, 0.4, 0.5, rep(0.5,n.true-5), rep(0.2,n.noise)))

y <- rbinom(ncalc, 1, invlogit(cbind(1,x) %*% beta))

data.calc <- data.frame(y,x)

fit <- glm(y ~ ., data = data.calc, family = 'binomial')

LR <- -2 * (as.numeric(logLik(glm(y ~ 1, data = data.calc,

family = binomial(link = "logit")))) -

as.numeric(logLik(fit)))

r2 <- 1 - exp(-LR/ncalc)

n_init <- round((n.predictors)/ ((S-1)*log(1-r2/S)))

n_init

n <- n_init}

# r2 <- as.numeric(approximate_R2(c_est, p, n = 2000000)[2])

# n_init <- round((n.predictors)/ ((S-1)*log(1-r2/S)))

# n_init

xval <-rmvbin(nval, margprob=c(0.7, 0.7, 0.3, 0.4, 0.5, rep(0.5,n.true-5), rep(0.2,n.noise)))

# yval <- rbinom(nval, 1, invlogit(cbind(1,xval) %*% beta))

for (i in 1: nsim){

set.seed(i)

x <- rmvbin(n, margprob=c(0.7, 0.7, 0.3, 0.4, 0.5, rep(0.5,n.true-5), rep(0.2,n.noise)))

y <- rbinom(n, 1, invlogit(cbind(1,x)%*%beta))

yval <- rbinom(nval, 1, invlogit(cbind(1,xval) %*% beta))

p_true <- as.vector(invlogit(cbind(1,xval) %*% beta))

#a <- fastglm(cbind(1,x), y, family=binomial())

a <- fastLR(cbind(1,x), y)

eta_est <- cbind(1, xval) %*% as.vector(a$coef)

p_est <- as.vector(invlogit(eta_est))

#fit <- fastglm(cbind(1,eta_est), yval, family=binomial())

fit <- fastLR(cbind(1,eta_est), yval )

cs[i] <- fit$coef[2]

mape[i] <- mean(abs(p_true-p_est))

}

#hist(cs, main = paste("CS=",round(median(cs,na.rm=TRUE)/0.0025)*0.0025, "N=",n))

df <- data.frame(cs)

pl <- ggplot(df, aes(x=cs)) +

geom_density() + ggtitle(paste("Target C = ", c, ", Target Prevalence = ", p,"\n", "N = ",n, ", Expected CS = ",round(median(cs,na.rm=TRUE)/0.005)*0.005,

", Expected MAPE=", round(median(mape,na.rm=TRUE),4), sep="")) +

geom_vline(aes(xintercept=median(cs)), color="blue", linetype="dashed", size=1)

print(pl)

df<- c(round(median(cs,na.rm=TRUE)/0.005)*0.005, sqrt(var(cs,na.rm=TRUE)), round(median(mape,na.rm=TRUE),4) )

xval <-rmvbin(1000000, margprob=c(0.7, 0.7, 0.3, 0.4, 0.5, rep(0.5, n.true-5), rep(0.2, n.noise)))

yval <- rbinom(1000000, 1, invlogit(cbind(1,xval)%*%beta))

set.seed(2022)

prev <- mean(yval)

cstat <- pROC::roc(as.vector(yval), as.vector(invlogit(cbind(1,xval)%*%beta)), quiet=TRUE)$auc

# cstat <- quickcstat(yval, invlogit(cbind(1,xval) %*% beta))

df <- data.frame(n, ceiling(mean(cs, na.rm = TRUE)/0.0025) * 0.0025,

round(sqrt(stats::var(cs,na.rm = TRUE)), 4),

round(sqrt( mean( ((cs-1)^2), na.rm=TRUE) ), 4),

round(mean(ifelse( (cs < 0.8), 1, 0),na.rm=TRUE), 2),

round(mean(mape, na.rm = TRUE),4),

round(sqrt(stats::var(mape,na.rm = TRUE)), 4),

round(prev, 3),

round(cstat, 3 ),

n.predictors)

names(df) <- c("N", "Mean_CS", "SD_CS", "RMSD_CS", "Pr(CS<0.8)", "Mean_MAPE", "SD_MAPE", "Prev.", "C-Stat.", " # Predictors")

performance <- df[,-3]

performance <- df

performance

}

expected_cs_binary_binarypred_r2riley <- function(n, S, p, c, beta = c(-0.5,-0.3,0.3,0.15,0.15) , n.true=5, n.noise=7, r2=0, nsim=500, nval=50000){

beta = c(-0.5,-0.3,0.3,0.15,0.15)

n.predictors <- n.true + n.noise

set.seed(2022)

beta <- adjust_multiplier_binary(c, p, beta = beta, n.true = n.true, n.noise = n.noise)

cs <- NULL

mape <- NULL

# xval <- rmvbin(1000000, margprob=c(0.7, 0.7, 0.3, 0.4, 0.5, rep(0.2,n.noise)))

# yval <- rbinom(1000000, 1, invlogit(cbind(1,xval)%*%beta))

# p_true <- invlogit(cbind(1,xval)%*%beta)

# p_est <- mean(yval)

# c_est <-quickcstat(yval,p_true)

# p_est

# c_est

# eta_est <- cbind(1,xval)%*%beta

# hist(eta_est)

#

MaxR2 <- 1-(((p^(p))*((1-p)^(1-p)))^2)

ncalc <- 2000000

x <- rmvbin(round(ncalc), margprob=c(0.7, 0.7, 0.3, 0.4, 0.5, rep(0.5,n.true-5), rep(0.2,n.noise)))

y <- rbinom(ncalc, 1, invlogit(cbind(1,x) %*% beta))

data.calc <- data.frame(y,x)

# fit <- glm(y ~ ., data = data.calc, family = 'binomial')

#

# LR <- -2 * (as.numeric(logLik(glm(y ~ 1, data = data.calc,

# family = binomial(link = "logit")))) -

# as.numeric(logLik(fit)))

# r2 <- 1 - exp(-LR/ncalc)

# n_init <- round((n.predictors)/ ((S-1)*log(1-r2/S)))

# n_init

# n <- n_init

# r2 <- as.numeric(approximate_R2(c_est, p, n = 2000000)[2])

# n_init <- round((n.predictors)/ ((S-1)*log(1-r2/S)))

# n_init

xval <-rmvbin(nval, margprob=c(0.7, 0.7, 0.3, 0.4, 0.5, rep(0.5,n.true-5), rep(0.2,n.noise)))

# yval <- rbinom(nval, 1, invlogit(cbind(1,xval) %*% beta))

for (i in 1: nsim){

set.seed(i)

x <- rmvbin(n, margprob=c(0.7, 0.7, 0.3, 0.4, 0.5, rep(0.5,n.true-5), rep(0.2,n.noise)))

y <- rbinom(n, 1, invlogit(cbind(1,x)%*%beta))

yval <- rbinom(nval, 1, invlogit(cbind(1,xval) %*% beta))

p_true <- as.vector(invlogit(cbind(1,xval) %*% beta))

#a <- fastglm(cbind(1,x), y, family=binomial())

a <- fastLR(cbind(1,x), y)

eta_est <- cbind(1, xval) %*% as.vector(a$coef)

p_est <- as.vector(invlogit(eta_est))

#fit <- fastglm(cbind(1,eta_est), yval, family=binomial())

fit <- fastLR(cbind(1,eta_est), yval )

cs[i] <- fit$coef[2]

mape[i] <- mean(abs(p_true-p_est))

}

#hist(cs, main = paste("CS=",round(median(cs,na.rm=TRUE)/0.0025)*0.0025, "N=",n))

df <- data.frame(cs)

pl <- ggplot(df, aes(x=cs)) +

geom_density() + ggtitle(paste("Target C = ", c, ", Target Prevalence = ", p,"\n", "N = ",n, ", Expected CS = ",round(median(cs,na.rm=TRUE)/0.005)*0.005,

", Expected MAPE=", round(median(mape,na.rm=TRUE),4), sep="")) +

geom_vline(aes(xintercept=median(cs)), color="blue", linetype="dashed", size=1)

print(pl)

df<- c(round(median(cs,na.rm=TRUE)/0.005)*0.005, sqrt(var(cs,na.rm=TRUE)), round(median(mape,na.rm=TRUE),4) )

xval <-rmvbin(1000000, margprob=c(0.7, 0.7, 0.3, 0.4, 0.5, rep(0.5, n.true-5), rep(0.2, n.noise)))

yval <- rbinom(1000000, 1, invlogit(cbind(1,xval)%*%beta))

set.seed(2022)

prev <- mean(yval)

cstat <- pROC::roc(as.vector(yval), as.vector(invlogit(cbind(1,xval)%*%beta)), quiet=TRUE)$auc

eta_est <- cbind(1,xval)%*%beta

df <- data.frame(eta_est)

pl <- ggplot(df, aes(x=eta_est)) + geom_histogram(binwidth=0.2) +

ggtitle(paste("Target C = ", c, ", Target Prevalence = ", p))

pl

df <- data.frame(n, ceiling(mean(cs, na.rm = TRUE)/0.0025) * 0.0025,

round(sqrt(stats::var(cs,na.rm = TRUE)), 4),

round(sqrt( mean( ((cs-1)^2), na.rm=TRUE) ), 4),

round(mean(ifelse( (cs < 0.8), 1, 0),na.rm=TRUE), 2),

round(mean(mape, na.rm = TRUE),4),

round(sqrt(stats::var(mape,na.rm = TRUE)), 4),

round(prev, 3),

round(cstat, 3 ),

n.predictors)

names(df) <- c("N", "Mean_CS", "SD_CS", "RMSD_CS", "Pr(CS<0.8)", "Mean_MAPE", "SD_MAPE", "Prev.", "C-Stat.", " # Predictors")

performance <- df[,-3]

performance <- df

performance

}

samplesizedev_binary_s_binary_pred <- function(S, p, c, beta = c(-0.5,-0.3,0.3,0.15,0.15) , n.true=5, n.noise=7, nval = 25000, nsim = 1000, parallel = TRUE, r2true=TRUE, acc=0.0025){

set.seed(2023)

beta = c(-0.5,-0.3,0.3,0.15,0.15)

n.predictors <- n.true + n.noise

set.seed(2022)

betan <- adjust_multiplier_binary(c, p, beta = beta, n.true = n.true, n.noise = n.noise)

# print("ok")

if (r2true==TRUE) {

# True R2

MaxR2 <- 1-(((p^(p))*((1-p)^(1-p)))^2)

ncalc <- 2000000

x <- rmvbin(ncalc, margprob=c(0.7, 0.7, 0.3, 0.4, 0.5, rep(0.5,n.true-5), rep(0.2,n.noise)))

y <- rbinom(ncalc, 1, invlogit(cbind(1,x)%*%betan))

data.calc <- data.frame(y,x)

fit <- glm(y ~ ., data = data.calc, family = 'binomial')

LR <- -2 * (as.numeric(logLik(glm(y ~ 1, data = data.calc,

family = binomial(link = "logit")))) -

as.numeric(logLik(fit)))

r2 <- 1 - exp(-LR/ncalc)

n_init <- round((n.predictors)/ ((S-1)*log(1-r2/S)))

n_init} else {

# Approximation R2

r2 <- as.numeric(approximate_R2(c, p, n = 2000000)[2])

n_init <- round((n.predictors)/ ((S-1)*log(1-r2/S)))

n_init

}

if (c<=0.7) {inflation_f <- 1.3 ; min.opt <- n_init*0.6}

if (c>0.7 & c<=0.8) {inflation_f <- 1.6 ; min.opt <- n_init}

if (c>0.8 & c<=0.85) {inflation_f <- 2.1 ; min.opt <- n_init}

if (c>0.85 & c<=0.9) {inflation_f <- 2.8 ; min.opt <- n_init}

max.opt <- inflation_f*n_init

min.opt <- round(min.opt)

max.opt <- round(max.opt)

tol = ceiling(round(n_init/200)/5) * 5

print("ok")

print("Optimisation Starting ~ 1 min left...")

s_est <- function(n, nsim=nsim){

s <- expected_s_n_binary_binarypred(n, S = S, betan = betan, p = p, c = c, n.true=n.true, n.noise=n.noise, nval = nval, nsim = nsim, parallel=parallel, acc = acc)

s[1] - S

}

n <- bisection(s_est, min.opt, max.opt, tol = tol, nsim = nsim, acc = acc)

if (n > 2100) tol = ceiling(round(n/400)/5) * 5 else tol = ceiling(round(n/200)/5)

n <- round(n/5)*5

size <- NULL

size$rvs1 <- as.vector(round(n_init))

size$actual <- as.vector(round(n))

# size$correct_to_nearest <- as.vector(tol)

size

}

########################################################################################

expected_s_n_binary_binarypred <- function(n, S, p, c, betan, n.true, n.noise, nsim, nval, parallel, acc){

n.predictors <- n.true + n.noise

set.seed(2023)

xval <- rmvbin(nval, margprob=c(0.7, 0.7, 0.3, 0.4, 0.5, rep(0.5,n.true-5), rep(0.2,n.noise)))

yval <- rbinom(nval, 1, invlogit(cbind(1,xval) %*% betan))

if (parallel==TRUE) {

cores <- parallel::detectCores()

cl <- parallel::makeCluster(cores[1]-2)} else

cl <- parallel::makeCluster(2)

doParallel::registerDoParallel(cl)

`%dopar%` <- foreach::`%dopar%`

`%do%` <- foreach::`%do%`

cs <- NULL

i <- 0

a <- foreach::foreach(i=1:nsim, .packages=c('mvtnorm','RcppNumerical', 'ggplot2', 'bindata' )) %dopar% {

set.seed(i)

invlogit<-function(x) 1/(1+exp(-x))

x <- rmvbin(n, margprob=c(0.7, 0.7, 0.3, 0.4, 0.5, rep(0.5,n.true-5), rep(0.2,n.noise)))

y <- rbinom(n, 1, invlogit(cbind(1,x)%*%betan))

yval <- rbinom(nval, 1, invlogit(cbind(1,xval) %*% betan))

#a <- fastglm(cbind(1,x), y, family=binomial())

a <- RcppNumerical::fastLR(cbind(1,x), y)

eta_est <- cbind(1, xval) %*% as.vector(a$coef)

#fit <- fastglm(cbind(1,eta_est), yval, family=binomial())

fit <- RcppNumerical::fastLR(cbind(1,eta_est), yval)

cs[i] <- fit$coef[2]

cs[i]

}

parallel::stopCluster(cl)

cs <- unlist(a)

#graphics::hist(cs, main = paste("CS=",round(mean(cs,na.rm=TRUE)/0.0025)*0.0025, "N=",n))

df <- data.frame(cs)

df <- stats::na.omit(df)

cs_plot <- ggplot2:: ggplot(df, ggplot2::aes(x = cs), size=12) +

ggplot2::geom_density() + ggplot2::ggtitle(paste("N = ", round(n), "p = ", round(mean(yval),2), ", Expected CS = ", round(mean(cs,na.rm=TRUE)/0.002)*0.002, ", SD(CS) = ", round(sqrt(stats::var(cs,na.rm=TRUE)),3))) +

ggplot2::geom_vline( ggplot2::aes(xintercept = mean(cs, na.rm = TRUE)), color="blue", linetype ="dashed", size = 1) +

ggplot2::ylab("Density") + ggplot2::theme(text = ggplot2::element_text(size = 13)) +

ggplot2::xlab("Calibration Slope")

if ( abs(mean(cs, na.rm=TRUE)- 0.9) > 0.0051) cs_plot <- cs_plot + ggplot2::geom_vline( ggplot2::aes(xintercept = 0.9), color="red", linetype ="dashed", size = 1)

print(cs_plot)

c(round(mean(cs,na.rm=TRUE)/acc)*acc, sqrt(stats::var(cs)/nsim))

}

########################################################################################

# p=0.2

# c=0.8

# nsim=1000

# nval=25000

# parallel=TRUE

# r2true=TRUE

# n.noise=7

# n.true=5

# expected_s_n_binary_binarypred (200, S=0.9, p=0.1, c=0.8, beta=betain, n.true=5, n.noise=7, nsim=100, nval=25000, parallel=TRUE)

# samplesizedev_binary_s_binary_pred(S=0.9, p=0.2, c=0.8, beta = c(-0.5,-0.3,0.3,0.15,0.15) , n.true=5, n.noise=7, nval = 25000, nsim = 1000, parallel = TRUE)

# samplesizedev_binary_s_binary_pred(S=0.9, p=0.1, c=0.7, beta = c(-0.5,-0.3,0.3,0.15,0.15) , n.true=5, n.noise=7, nval = 25000, nsim = 1000, parallel = TRUE)

expected_optr2_binary_corr <- function(n, p, c, beta = rep(1/n.predictors, n.predictors), n.predictors, nsim = 1000, nval = 25000, cor0=0, cor1=0, method ="MLE", parallel=TRUE, long=TRUE, r2true=TRUE){

# Find mean and variance for Normal linear predictor

beta = c(c(0.4, 0.2, 0.2, 0.1, 0.1), rep(0,7))

set.seed(2023)

mean_var <- find_mu_sigma(p, c, tol = 0.0001)

mean <- mean_var[1]

variance <- mean_var[2]

# Find beta that corresponds to that variance

if (cor0==0 & cor1 ==0) {

beta <- beta * sqrt(mean_var[2]/sum(beta^2))

sigma <- diag(1, n.predictors)} else

{

beta <- adjust_multiplier_correlated(c=c, mean = mean, beta = beta, n.predictors = n.predictors, cor0=cor0, cor1=cor1)

beta

n.noise <- length(beta[beta==0])

n.true <- n.predictors-n.noise

# Specify correlation matrix

sigma <- matrix(0, nrow = n.predictors, ncol = n.predictors)

sigma[1:n.true, 1:n.true] <- cor0

if (n.noise>0) {

sigma[(n.true+1):n.predictors, (n.true+1):n.predictors] <- cor1}

diag(sigma) <- 1

}

if (r2true==TRUE) {

# True R2

ncalc <- 2000000

x <- mvtnorm::rmvnorm(ncalc, rep(0, n.predictors), sigma = sigma )

y <- stats::rbinom( ncalc, 1, invlogit(mean + x%*%beta))

p <- mean(y)

MaxR2 <- 1-(((p^(p))*((1-p)^(1-p)))^2)

data.calc <- data.frame(y,x)

fit <- glm(y ~ ., data = data.calc, family = 'binomial')

LR <- -2 * (as.numeric(logLik(glm(y ~ 1, data = data.calc,

family = binomial(link = "logit")))) -

as.numeric(logLik(fit)))

r2 <- 1 - exp(-LR/ncalc)

L0 <- as.numeric(logLik(glm(y ~ 1, data = data.calc,

family = binomial(link = "logit"))))

# For optimism

S <- r2 /(r2+0.05*MaxR2 )

n_init <- round((n.predictors)/ ((S-1)*log(1-r2/S)))

n <- n_init

n_rvs1 <- round((n.predictors)/ ((0.9-1)*log(1-r2/0.9)))

# pmsampsize(type = "b", rsquared = r2, parameters = 12, prevalence = 0.1)

}

r2_cs_true <- r2

n

# Approximate R2

# r2 <- as.numeric(approximate_R2(c_est, p, n = 2000000)[2])

# n_init <- round((n.predictors)/ ((S-1)*log(1-r2/S)))

# n_init

# xval <- mvtnorm::rmvnorm(nval, rep(0, n.predictors), sigma = sigma)

#yval <- stats::rbinom(nval, 1, invlogit(mean + xval%*%beta))

if (parallel==TRUE) {

cores <- parallel::detectCores()

cl <- parallel::makeCluster(cores[1]-2)} else

cl <- parallel::makeCluster(2)

doParallel::registerDoParallel(cl)

`%dopar%` <- foreach::`%dopar%`

`%do%` <- foreach::`%do%`

opt <- NULL

i <- 0

a<- foreach::foreach(i = 1:nsim, .packages=c('mvtnorm','RcppNumerical', 'ggplot2' )) %dopar% {

set.seed(i)

invlogit <- function(x) 1/(1+exp(-x))

x <- mvtnorm::rmvnorm(round(n), rep(0, n.predictors), sigma = sigma )

y <- stats::rbinom(round(n), 1, invlogit(mean + x%*%beta))

data.calc <- data.frame(y,x)

fit <- glm(y ~ ., data = data.calc, family = 'binomial')

LR <- -2 * (as.numeric(logLik(glm(y ~ 1, data = data.calc,

family = binomial(link = "logit")))) -

as.numeric(logLik(fit)))

L0 <- as.numeric(logLik(glm(y ~ 1, data = data.calc,

family = binomial(link = "logit"))))

MaxR2_app = (1 - exp(2*L0/n))

r2_cs_app <- 1 - exp(-LR/n)

r2_cs_true/ r2_cs_app

opt[i] <- r2_cs_app/MaxR2 - r2_cs_true/MaxR2

# opt[i] <- r2_cs_app

# opt[i] <- 1 - n.predictors/LR

c(opt[i])

}

parallel::stopCluster(cl)

b <- matrix(unlist(a), byrow=TRUE, nrow=nsim)

# opt <- b[,1]

# opt <- median(b[,1])/MaxR2 - r2_cs_true/MaxR2

# opt <- median(b[,1])

opt <- mean(b[,1])

# r2_cs_app <- 1 - exp(-mean(opt)/n)

set.seed(2022)

xval <- mvtnorm::rmvnorm(2000000, rep(0,n.predictors), sigma = sigma)

yval <- stats::rbinom(2000000, 1, invlogit(mean + xval %*% beta))

prev <- mean(yval)

# cstat <- quickcstat(yval, invlogit(mean + xval %*% beta))

cstat <- pROC::roc(as.vector(yval), invlogit(mean + xval %*% beta), quiet=TRUE)$auc

# Short format for presentation

df <- data.frame(n, n_rvs1,

round(mean(opt, na.rm = TRUE),4),

round(prev, 4),

round(cstat, 4 ),

n.predictors)

names(df) <- c("N", "N_rvs1", "Mean_Opt", "Prev.", "C-Stat.", " # Predictors")

performance <- df[,-3]

performance1 <- df

# Long format for simulations

performance2 <- data.frame(n, n_rvs1, n.predictors, round(cstat,3), round(prev, 3), opt)

names(performance2) = c("n", "n_rvs1", "npred", "cstat","prev","opt_est")

performance2

if (long==TRUE) performance <- performance2 else performance <- performance1

performance

}

expected_overall_binary_corr <- function(n, p, c, beta = rep(1/n.predictors, n.predictors), n.predictors, nsim = 1000, nval = 25000, cor0=0, cor1=0, method ="MLE", parallel=TRUE, long=TRUE, r2true=FALSE){

beta = c(c(0.4, 0.2, 0.2, 0.1, 0.1), rep(0,7))

set.seed(2023)

# Find mean and variance for Normal linear predictor

mean_var <- find_mu_sigma(p, c, tol = 0.0001)

mean <- mean_var[1]

variance <- mean_var[2]

# Find beta that corresponds to that variance

if (cor0==0 & cor1 ==0) {

beta <- beta * sqrt(mean_var[2]/sum(beta^2))

sigma <- diag(1, n.predictors)} else

{

beta <- adjust_multiplier_correlated(c=c, mean = mean, beta = beta, n.predictors = n.predictors, cor0=cor0, cor1=cor1)

beta

n.noise <- length(beta[beta==0])

n.true <- n.predictors-n.noise

# Specify correlation matrix

sigma <- matrix(0, nrow = n.predictors, ncol = n.predictors)

sigma[1:n.true, 1:n.true] <- cor0

if (n.noise>0) {

sigma[(n.true+1):n.predictors, (n.true+1):n.predictors] <- cor1}

diag(sigma) <- 1

}

n <- ceiling((1.96/0.05)^2*p*(1-p))

if (parallel==TRUE) {

cores <- parallel::detectCores()

cl <- parallel::makeCluster(cores[1]-2)} else

cl <- parallel::makeCluster(2)

doParallel::registerDoParallel(cl)

`%dopar%` <- foreach::`%dopar%`

`%do%` <- foreach::`%do%`

error <- NULL

i <- 0

xval <- mvtnorm::rmvnorm(2000000, rep(0, n.predictors), sigma = sigma)

yval <- stats::rbinom(2000000, 1, invlogit(mean + xval %*% beta))

prev <- mean(yval)

a<- foreach::foreach(i = 1:nsim, .packages=c('mvtnorm','RcppNumerical', 'ggplot2' )) %dopar% {

set.seed(i)

invlogit <- function(x) 1/(1+exp(-x))

x <- mvtnorm::rmvnorm(round(n), rep(0, n.predictors), sigma = sigma )

y <- stats::rbinom(round(n), 1, invlogit(mean + x%*%beta))

x <- scale(x, scale=FALSE)

mean(y)

data.calc <- data.frame(y,x)

# a <- RcppNumerical::fastLR(as.matrix(rep(1,n)), y)

# a <- RcppNumerical::fastLR(cbind(1,x), y)

# a$coef[1]

fit <- glm(y ~ ., data = data.calc, family = 'binomial')

fit$coef[1]

p_est <- invlogit(as.vector(fit$coef)[1])

p_est

x_est<-c(1, rep(0,12))

xb <- t(x_est)%*%fit$coef

var = dlogis(xb) %*%t(x_est) %*% vcov(fit) %*% x_est %*% dlogis(xb)

error[i] = 1.96 * sqrt(var)

# error[i] <- 1.96*sqrt(p_est*(1-p_est)/n)

# SE <- sqrt(p_est*(1-p_est)/n)

# error[i] <- ifelse( ( (prev >= (p_est-qnorm(.975)*SE) ) & (prev <= (p_est+qnorm(.975)*SE) ) ) ,1,0)

c(error[i])

}

parallel::stopCluster(cl)

b <- matrix(unlist(a), byrow=TRUE, nrow=nsim)

error <- mean(b[,1])

set.seed(2022)

xval <- mvtnorm::rmvnorm(2000000, rep(0,n.predictors), sigma = sigma)

yval <- stats::rbinom(2000000, 1, invlogit(mean + xval %*% beta))

prev <- mean(yval)

# cstat <- quickcstat(yval, invlogit(mean + xval %*% beta))

cstat <- pROC::roc(as.vector(yval), invlogit(mean + xval %*% beta), quiet=TRUE)$auc

# Short format for presentation

df <- data.frame(n,

round(mean(error, na.rm = TRUE),4),

round(prev, 4),

round(cstat, 4 ),

n.predictors)

names(df) <- c("N", "Mean_Error", "Prev.", "C-Stat.", " # Predictors")

performance <- df[,-3]

performance1 <- df

# Long format for simulations

performance2 <- data.frame(n, n.predictors, round(cstat,3), round(prev, 3), error)

names(performance2) = c("n", "npred", "cstat","prev","error_est")

performance2

if (long==TRUE) performance <- performance2 else performance <- performance1

performance

}

#### Expected CS and slope for skewed predictors

expected_cs_mape_binary_skewed <- function(n, p, c, n.predictors=12, nsim = 1000, nval = 25000) {

# if (n==0) {

# size <- samplesizedev(outcome="Binary", S = 0.9, phi = 0.1, c = c, p = 12, parallel = TRUE)

# n

# n <- size$sim

# }

# nval <- 100000

# nsim=2000

# c=0.7

# nsim=2000

set.seed(2023)

x1 <- rexp(nval,0.3)

x2 <- rexp(nval,0.3)

x3 <- rexp(nval,0.3)

x4 <- rexp(nval,0.3)

x5 <- rbinom(nval,1, 0.2)

x2 <- rexp(nval,0.3)

x6 <- rexp(nval,0.3)

x7 <- rexp(nval,0.3)

x8 <- rexp(nval,0.3)

x9 <- rexp(nval,0.3)

x10 <- rexp(nval,0.3)

x11 <- rexp(nval,0.3)

x12 <- rexp(nval,0.3)

xval<-cbind(x1,x2,x3,x4,x5, x6,x7,x8,x9,x10, x11, x12)

xval<-scale(xval)

if (c==0.65) {b0=-2.3 ; f=0.73}

if (c==0.7) {b0=-2.4 ; f=0.98}

if (c==0.75) {b0=-2.52 ; f=1.26}

if (c==0.8) {b0=-2.7; f=1.6}

if (c==0.85) {b0=-2.95; f=2.03}

if (c==0.9) {b0=-3.35; f=2.63}

b <- c(b0,f*rep(0.32,5), rep(0,7))

etaval<- cbind(1,xval)%*%b

# etaval<- cbind(1,xval)%*%b

#

# yval <- stats::rbinom(round(nval), 1, invlogit(etaval))

# mean(yval)

# hist(etaval)

# p_true <- as.vector(invlogit(etaval))

# quickcstat(yval, invlogit(etaval))

cores <- parallel::detectCores()

cl <- parallel::makeCluster(cores[1]-4)

doParallel::registerDoParallel(cl)

`%dopar%` <- foreach::`%dopar%`

`%do%` <- foreach::`%do%`

cs <- NULL

mape <- NULL

a<- foreach::foreach(i = 1:nsim, .packages=c('mvtnorm','RcppNumerical', 'ggplot2' )) %dopar% {

# for (i in 1:nsim) {

invlogit <- function(x) 1/(1+exp(-x))

set.seed(i)

x1 <- rexp(n,0.3)

x2 <- rexp(n,0.3)

x3 <- rexp(n,0.3)

x4 <- rexp(n,0.3)

x5 <- rbinom(n,1, 0.2)

x2 <- rexp(n,0.3)

x6 <- rexp(n,0.3)

x7 <- rexp(n,0.3)

x8 <- rexp(n,0.3)

x9 <- rexp(n,0.3)

x10 <- rexp(n,0.3)

x11 <- rexp(n,0.3)

x12 <- rexp(n,0.3)

x<-cbind(x1,x2,x3,x4,x5, x6,x7,x8,x9,x10, x11, x12)

x<-scale(x)

if (c==0.65) {b0=-2.3 ; f=0.73}

if (c==0.7) {b0=-2.4 ; f=0.98}

if (c==0.75) {b0=-2.52 ; f=1.26}

if (c==0.8) {b0=-2.7; f=1.6}

if (c==0.85) {b0=-2.95; f=2.03}

if (c==0.9) {b0=-3.35; f=2.63}

b <- c(b0,f*rep(0.32,5), rep(0,7))

eta <- cbind(1,x)%*%b

etaval <- cbind(1,xval)%*%b

y <- stats::rbinom(round(n), 1, invlogit(eta))

yval <- stats::rbinom(round(nval), 1, invlogit(etaval))

p_true <- as.vector(invlogit(etaval))

a <- RcppNumerical::fastLR(cbind(1,x), y)

eta_est <- cbind(1, xval) %*% as.vector(a$coef)

p_est <- as.vector(invlogit(eta_est))

fit <- RcppNumerical::fastLR(cbind(1,eta_est), yval, start = c(0,0.9) )

cs[i] <- fit$coef[2]

mape[i] <- mean(abs(p_true-p_est))

c(cs[i], mape[i])

}

bb <- matrix(unlist(a), byrow=TRUE, nrow=nsim)

cs <- bb[,1]

mape <- bb[,2]

df <- data.frame(n, ceiling(mean(cs, na.rm = TRUE)/0.0025) * 0.0025,

round(sqrt(stats::var(cs,na.rm = TRUE)), 4),

round(sqrt( mean( ((cs-1)^2), na.rm=TRUE) ), 4),

round(mean(ifelse( (cs < 0.8), 1, 0),na.rm=TRUE), 2),

round(mean(mape, na.rm = TRUE),4),

round(sqrt(stats::var(mape,na.rm = TRUE)), 4),

round(p, 4),

round(c, 4 ),

n.predictors)

names(df) <- c("N", "Mean_CS", "SD_CS", "RMSD_CS", "Pr(CS<0.8)", "Mean_MAPE", "SD_MAPE", "Prev.", "C-Stat.", " # Predictors")

# performance <- df[,-3]

#performance

df

}

####################################################################################################################################

# Part B: Run the code below for the main simulation results

############################################################

# Set the working directory to point to folder 'Simulation'

# Create this folder first to save the results and graphs (if needed)

# To save figures create the subfolder “plots development"

# setwd("yourpath\\Simulation")

# Install packages if not already installed

require(MASS); require(doParallel); require(rms);

require(speedglm); require(tidyverse);require(apricom);require(patchwork);

require(logistf); require(pmsampsize); require(plyr); require(pROC);

require(blorr); require(DescTools); require(ggpubr);require(predtools)

require(brglm2); require(devtools); require(DescTools)

require(ggplot2); require(dplyr); require(glmnet);

require(openxlsx);require(forcats); require(plyr);require(dplyr)

require(bindata);require(RcppNumerical); require(foreach)

rm(list=ls())

es <- NULL

es_sd <- NULL

es_rmsd <- NULL

es_pr <- NULL

mape <- NULL

mape_sd <- NULL

prev <- NULL

cstat <- NULL

method <- NULL

n.predictors <- 12

nval <- 100000

i<-0

sumcin <- NULL

sumc <- NULL

sump <- NULL

#Prevalence

for (p in c(0.1,0.3,0.5)) {

#c-statistic

for (c in c(0.65, 0.7, 0.75, 0.8, 0.85, 0.9)) {

i<-i+1

system.time (n <- samplesizedev_binary_s_corr(S = 0.9, p = p, c = c, n.predictors = n.predictors, nsim = 2000, nval=nval, parallel = TRUE, beta = c(c(0.4, 0.2, 0.2, 0.1, 0.1), rep(0,7)), cor0=0.1, cor1=0.05, acc=0.0025) )

a <- expected_cs_mape_binary_corr(n = n$rvs1, p = p, c = c, n.predictors = n.predictors, nsim = 2000, parallel = TRUE, nval=nval, beta = c(c(0.4, 0.2, 0.2, 0.1, 0.1), rep(0,7)), cor0=0.1, cor1=0.05, r2 = FALSE)

b <- expected_cs_mape_binary_corr(n = n$actual, p = p, c = c, n.predictors = n.predictors, nsim = 2000, parallel = TRUE, nval=nval, beta = c(c(0.4, 0.2, 0.2, 0.1, 0.1), rep(0,7)), cor0=0.1, cor1=0.05, r2=FALSE)

sumcin <- rbind( cbind(as.matrix(a),0,1), cbind(as.matrix(b),1,n$actual/n$rvs1))

names(sumcin) <- c("n", "npred", "cstat","prev","cs_est","mape_est","cstat_est", "method", "inflation")

sumc<-rbind(sumc, sumcin)

}

sump <- rbind(sump, sumc)

}

long_res <- data.frame(sump)

names(long_res) <- c("n", "npred", "cstat","prev","cs_est","mape_est","cstat_est", "method", "inflation" )

# Prepare for plotting

long_res$prev <- round(long_res$prev,2)

long_res$cstat <- round(long_res$cstat,2)

long_res$inflation[long_res$inflation==1]=NA

long_res$inflation <- round(long_res$inflation,2)

# Create additional measures

long_res$int_cs <- ifelse( (long_res$cs_est >= 0.9 & long_res$cs_est <=1.1), 1, 0)

long_res$int_cs <- ifelse( (long_res$cs_est < 0.8), 1, 0)

long_res$int_cs2 <- ifelse( (long_res$cs_est < 0.9), 1, 0)

long_res$int_c <- ifelse( ((long_res$cstat_est >= long_res$cstat-0.02) ), 1, 0)

long_res$int_c2 <- ifelse( ((long_res$cstat_est >= long_res$cstat-0.025) ), 1, 0)

# Aggregate per scenario and method (RvS-1, By simulation)

short_res <- ddply(long_res, .(cstat, prev, method), function(df) {

data.frame(count = nrow(df),

n = mean(df$n, na.rm = T),

npred = mean(df$npred, na.rm = T),

cstat = mean(df$cstat, na.rm = T),

prev = mean(df$prev, na.rm = T),

method = mean(df$method, na.rm = T),

cs = mean(df$cs_est, na.rm = T),

cs_sd = var(df$cs_est, na.rm = T),

int_cs = mean(df$int_cs, na.rm = T),

int_cs2 = mean(df$int_cs2, na.rm = T),

mape = mean(df$mape_est, na.rm = T),

cstat_est = mean(df$cstat_est, na.rm = T),

int_c = mean(df$int_c, na.rm = T),

int_c2 = mean(df$int_c2, na.rm = T),

inflation = mean(df$inflation, na.rm = T)

)

})[,-1]

short_res$method = factor(short_res$method)

levels(short_res$method) = c("RvS-1", "By Simulation")

short_res

long_res$method = factor(long_res$method)

levels(long_res$method) = c("RvS-1", "By Simulation")

long_res[1,]

res <- short_res

res$epv <- round(res$n*res$prev/res$npred,2)

res

prev_name<- list(

'01'="Prev=0.1",

'03'="Prev=0.3",

'05'='Prev=0.5'

)

c_name<- list(

'0.65'="C=0.65",

'0.70'="C=0.70",

'0.75'="C=0.75",

'0.80'="C=0.80",

'0.85'='C=0.85',

'0.90'='C=0.90'

)

c_labeller <- function(variable,value){

return(c_name[value])

}

prev_labeller <- function(variable,value){

return(prev_name[value])

}

res$cstat = factor(res$cstat)

res$prev = factor(res$prev)

long_res$cstat = factor(long_res$cstat)

long_res$prev = factor(long_res$prev)

# save.image("development_rvs1.Rdata")

# load("development_rvs1.Rdata")

res%>%ggplot(aes(cstat, cs_sd, group=prev,col=prev))+ geom_line(size=1) + geom_line(size=1) +

facet_wrap(~method) +

ylab("Variance of the Calibration Slope over 2000 sims") + xlab("True c-statistic") + theme_bw() +

theme(legend.position="bottom") +

labs(shape="Prevalence", colour="Prevalence") + theme(text = element_text(size = 14))

cs_plot_rvs1 <- ggplot(res%>%filter(method=="RvS-1"), aes(x = cstat, y = cs, group=prev, col=prev)) + geom_line(size=1) +

ylab("Calibration Slope (mean over 2000 simulations)") + xlab("True c-statistic") + theme_bw() +

theme(legend.position="bottom") +

geom_hline(yintercept = 0.9, linetype="dashed",size=1) + labs(colour=res$prev)+ scale_y_continuous(breaks=, limits = c(0.75, 0.95)) +

labs(shape="Prevalence", colour="Prevalence") + theme(text = element_text(size = 14))

cs_plot_rvs1

ggsave("plots development//rvs1_mean_cs.pdf", width=8, height=6)

ggsave("plots development//rvs1_mean_cs.png", width=8, height=6, dpi=600)

ggsave("plots development//figure_1.png", width=8, height=6, dpi=600)

cs_boxplot_rvs1 <- ggplot(long_res %>% filter(method=='RvS-1'), aes(x=as.factor(cstat), y=cs_est, fill=prev)) +

geom_hline(yintercept=0.9,linetype="dashed") +

geom_hline(yintercept=1.1,linetype="dashed") +

geom_boxplot() + ylab("Calibration Slope (2000 simulations)")+ xlab("True c-statistic")+

theme_bw()+

theme(legend.position="bottom")+

scale_y_continuous(breaks=c(0.6, 0.7,0.8, 0.9, 1, 1.1, 1.2, 1.3)) +

labs(colour=long_res$prev) +

labs(fill="Prevalence", colour="Prevalence") + theme(text = element_text(size = 14))

cs_boxplot_rvs1

ggsave("plots development//rvs1_cs_boxplots.pdf", width=8, height=6)

ggsave("plots development//rvs1_cs_boxplots.png", width=8, height=6, dpi=600)

ggsave("plots development//figure_s1.png", width=8, height=6, dpi=600)

cs_boxplot_simulation <- ggplot(long_res %>% filter(method=='By Simulation'), aes(x=as.factor(cstat), y=cs_est, fill=prev)) +

geom_hline(yintercept=0.9,linetype="dashed") +

geom_hline(yintercept=1.1,linetype="dashed") +

geom_boxplot() + ylab("Calibration Slope (2000 simulations)")+ xlab("True c-statistic")+

theme_bw()+

theme(legend.position="bottom")+

scale_y_continuous(breaks=c(0.6, 0.7,0.8, 0.9, 1, 1.1, 1.2, 1.3)) +

labs(colour=long_res$prev) +

labs(fill="Prevalence", colour="Prevalence") + theme(text = element_text(size = 14))

cs_boxplot_simulation

size_plot_rvs1 <- res%>%ggplot(aes(cstat, n, group=method,col=method))+ geom_line(size=1) +

facet_wrap(~prev,labeller=prev_labeller) +

geom_text(aes(y = max(n)+500, label=round(inflation,2)), color = "black", fontface = 2, size = 4.5) +

theme_bw() + theme(legend.position="bottom") +

ylab("Sample size to achieve expected CS=0.9 (log-scale)") + theme_bw()+

theme(legend.position="bottom")+

xlab("True c-statistic") +

labs(shape="Method", colour="Method") + theme(text = element_text(size = 14))

# +scale_y_continuous(trans='log10')

size_plot_rvs1

ggsave("plots development//rvs1_size_req_cs.pdf", width=13, height=6)

ggsave("plots development//rvs1_size_req_cs.png", width=13, height=6)

epv_plot_rvs1 <- res%>%ggplot(aes(cstat, epv, group=method,col=method))+ geom_line(size=1) +

facet_wrap(~prev,labeller=prev_labeller) +

geom_text(aes(y = max(epv)+10, label=round(inflation,2)), color = "black", fontface = 2, size = 4.5) +

scale_y_continuous(limits = c(0, 75)) +

ylab("EPV to achieve expected CS=0.9") + theme_bw()+

theme(legend.position="bottom") +

xlab("True c-statistic") +

labs(shape="Method", colour="Method") + theme(text = element_text(size = 14))

epv_plot_rvs1

ggsave("plots development//rvs1_epv_req_cs.pdf", width=13, height=6)

ggsave("plots development//rvs1_epv_req_cs.png", width=13, height=6, dpi=600)

ggsave("plots development//figure_2.png", width=13, height=6, dpi=600)

cs_int_plot_rvs1 <- res%>%ggplot(aes(cstat, int_cs, group=method,col=method))+ geom_line(size=1) +

scale_y_continuous(limits = c(0, 0.7)) +

facet_wrap(~prev,labeller=prev_labeller) +

ylab("Proportion of times CS<0.8") + theme_bw()+

theme(legend.position="bottom") +

xlab("True c-statistic") +

labs(shape="Method", colour="Method") + theme(text = element_text(size = 14))

res%>%ggplot(aes(cstat, int_cs2, group=method,col=method))+ geom_line(size=1) +

scale_y_continuous(limits = c(0, 1)) +

facet_wrap(~prev,labeller=prev_labeller) +

ylab("Proportion of times CS<0.9") + theme_bw()+

theme(legend.position="bottom") +

xlab("True c-statistic") +

labs(shape="Method", colour="Method") + theme(text = element_text(size = 14))

cs_int_plot_rvs1

ggsave("plots development//rvs1_cs_interval08.pdf", width=8, height=5)

ggsave("plots development//rvs1_cs_interval08.png", width=8, height=5)

ggsave("plots development//figure_3.png", width=8, height=5, dpi=600)

c_int_plot_rvs1 <- res%>%ggplot(aes(cstat, int_c, group=method,col=method))+ geom_line(size=1) +

scale_y_continuous(limits = c(0.6, 1)) +

facet_wrap(~prev,labeller=prev_labeller) +

ylab("Proportion of times c-stat within 0.02 of the maximum") + theme_bw()+

theme(legend.position="bottom") +

xlab("True c-statistic") +

labs(shape="Method", colour="Method") + theme(text = element_text(size = 14))

c_int_plot_rvs1

ggsave("plots development//rvs1_c_interval.pdf", width=8, height=5)

ggsave("plots development//rvs1_c_interval.png", width=8, height=5)

ggsave("plots development//figure_s2.png", width=8, height=5, dpi=600)

res%>%ggplot(aes(cstat, int_c2, group=method,col=method))+ geom_line(size=1) +

scale_y_continuous(limits = c(0.7, 1)) +

facet_wrap(~prev,labeller=prev_labeller) +

ylab("Proportion of times c-stat within 0.025 of the maximum") + theme_bw()+

theme(legend.position="bottom") +

xlab("True c-statistic") +

labs(shape="Method", colour="Method") + theme(text = element_text(size = 14))

View(res)

res_print <-cbind(res%>%filter(method=="By Simulation")%>%select(cstat, prev, n, epv, inflation),

res%>%filter(method=="RvS-1")%>%select(n, epv))

res_print <- arrange(res_print, prev, cstat)

names(res_print)=c("cstat", "prev","n_sim", "epv_sim","inflation","n_rvs1","epv_rvs1")

res_print<- select(res_print, prev, cstat, epv_rvs1, epv_sim, n_rvs1, n_sim, inflation)

res_print$n_rvs1 <- round(res_print$n_rvs1/10)*10

res_print$epv_rvs1 <- round(res_print$epv_rvs1,1 )

res_print$n_sim <- round(res_print$n_sim/10)*10

res_print$epv_sim <- round(res_print$epv_sim, 1)

View(res_print)

wb <- createWorkbook()

addWorksheet(wb, "Table Shrinkage")

writeData(wb, 1, res_print)

addFilter(wb, 1, row = 1, cols = 1:ncol(res_print))

saveWorkbook(wb, file = "Table_shrinkage.xlsx", overwrite = TRUE)

saveWorkbook(wb, file = "table_s1.xlsx", overwrite = TRUE)

# test<-short_res%>%filter(method=="By Simulation")%>%arrange(prev,cstat)

# test$n

###############################

# MAPE (RvS-2)

n.predictors <- 12

i<-0

sumcin <- NULL

sumc <- NULL

sump <- NULL

for (p in c(0.1, 0.3, 0.5)) {

for (c in c(0.65, 0.7, 0.75, 0.8, 0.85, 0.9)) {

i<-i+1

system.time (n <- samplesizedev_binary_mape_corr(MAPE = 0.025, p = p, c = c, n.predictors = n.predictors, nsim = 2000, nval=100000, parallel = TRUE, beta = c(c(0.4, 0.2, 0.2, 0.1, 0.1), rep(0,7)), cor0=0.1, cor1=0.05) )

# expected performance for RvS-2

a <- expected_cs_mape_binary_corr(n = n$rvs2, p = p, c = c, n.predictors = n.predictors, nsim = 2000, parallel = TRUE, nval=100000, beta = c(c(0.4, 0.2, 0.2, 0.1, 0.1), rep(0,7)), cor0=0.1, cor1=0.05)

# expected performance for actual size required

b <- expected_cs_mape_binary_corr(n = round(n$actual), p = p, c = c, n.predictors = n.predictors, nsim = 2000, parallel = TRUE, nval=100000, beta = c(c(0.4, 0.2, 0.2, 0.1, 0.1), rep(0,7)), cor0=0.1, cor1=0.05)

sumcin <- rbind( cbind(as.matrix(a),0,1), cbind(as.matrix(b),1,n$actual/n$rvs2))

sumc<-rbind(sumc, sumcin)

}

sump <- rbind(sump, sumc)

}

long_res <- data.frame(sump)

names(long_res) <- c("n", "npred", "cstat","prev","cs_est","mape_est","cstat_est", "method", "inflation" )

# Prepare for plotting

long_res$mape_per <- long_res$mape_est/ long_res$prev

long_res$prev <- round(long_res$prev,2)

long_res$cstat <- round(long_res$cstat,2)

long_res$inflation[long_res$inflation==1]=NA

long_res$inflation <- round(long_res$inflation,2)

data2 <- cbind(unique(long_res$prev), unique(as.numeric(long_res$prev))/10)

data2 <-data.frame(data2)

# Create additional measures

long_res$int_cs <- ifelse( (long_res$cs_est >= 0.9 & long_res$cs_est <=1.1), 1, 0)

long_res$int_cs <- ifelse( (long_res$cs_est < 0.8), 1, 0)

long_res$int_c <- ifelse( ((long_res$cstat_est >= long_res$cstat-0.02) ), 1, 0)

# Aggregate data - one row pr scenario and method (RvS-2, by sinulation)

short_res <- ddply(long_res, .(cstat, prev, method), function(df) {

data.frame(count = nrow(df),

n = mean(df$n, na.rm = T),

npred = mean(df$npred, na.rm = T),

cstat = mean(df$cstat, na.rm = T),

prev = mean(df$prev, na.rm = T),

method = mean(df$method, na.rm = T),

cs = mean(df$cs_est, na.rm = T),

int_cs = mean(df$int_cs, na.rm = T),

mape = mean(df$mape_est, na.rm = T),

mape_per = mean(df$mape_per, na.rm = T),

cstat_est = mean(df$cstat_est, na.rm = T),

int_c = mean(df$int_c, na.rm = T),

inflation = mean(df$inflation, na.rm = T)

)

})[,-1]

short_res$method = factor(short_res$method)

levels(short_res$method) = c("RvS-2", "By Simulation")

long_res$method = factor(long_res$method)

levels(long_res$method) = c("RvS-2", "By Simulation")

# save.image("development_rvs2.Rdata")

# load("development_rvs2.Rdata")

#Prepare for plotting

res <- short_res

res$epv <- round(res$n*res$prev/res$npred,2)

res

prev_name<- list(

'01'="Prev=0.1",

'03'="Prev=0.3",

'05'='Prev=0.5'

)

c_name<- list(

'0.65'="C=0.65",

'0.70'="C=0.70",

'0.75'="C=0.75",

'0.80'="C=0.80",

'0.85'='C=0.85',

'0.90'='C=0.90'

)

c_labeller <- function(variable,value){

return(c_name[value])

}

prev_labeller <- function(variable,value){

return(prev_name[value])

}

res$cstat = factor(res$cstat)

res$prev = factor(res$prev)

long_res$cstat = factor(long_res$cstat)

long_res$prev = factor(long_res$prev)

# Plots in Main paper and Appendix for main simulation

# MAPE lineplot

mape_plot_rvs2 <- ggplot(res%>%filter(method=="RvS-2"), aes(x = cstat, y = mape, group=prev, col=prev)) + geom_line(size=1) +

ylab("Mean MAPE (2000 simulations)") + xlab("True c-statistic") +

theme_bw() + theme(legend.position="bottom") +

geom_hline(data=data2, aes(yintercept=X2, color=factor(X1)), linetype="dashed", size=1) + labs(colour=res$prev)+

labs(shape="Prevalence", colour="Prevalence") + theme(text = element_text(size = 14)) +

scale_y_continuous(breaks=c(0, 0.01, 0.02, 0.03, 0.04, 0.05), limits = c(0, 0.055))

mape_plot_rvs2

# mape_plot_rvs2 <- ggplot(res%>%filter(method=="RvS-2"), aes(x = cstat, y = mape_per, group=prev, col=prev)) + geom_line(size=1) +

# ylab("Mean MAPE/prevalence (2000 simulations)") + xlab("True c-statistic") +

# theme_bw() + theme(legend.position="bottom") +

# geom_hline(yintercept = c(0.1), linetype="dashed", size=1) + labs(colour=res$prev)+

# labs(shape="Prevalence", colour="Prevalence") + theme(text = element_text(size = 14))

#

# mape_plot_rvs2

ggsave("plots development//rvs2_mean_mape.pdf", width=8, height=6)

ggsave("plots development//rvs2_mean_mape.png", width=8, height=6)

ggsave("plots development//figure_4.png", width=8, height=6, dpi=600)

mape_plot_rvs2 <- ggplot(res%>%filter(method=="RvS-2"), aes(x = cstat, y = mape, group=prev, col=prev)) + geom_line(size=1) +

ylab("Mean MAPE (2000 simulations)") + xlab("True c-statistic") +

theme_bw() + theme(legend.position="bottom") +

geom_hline(yintercept = c(0.01, 0.03, 0.05), linetype="dashed",size=1) + labs(colour=res$prev)+

labs(shape="Prevalence", colour="Prevalence") + theme(text = element_text(size = 14)) +

scale_y_continuous(breaks=c(0, 0.01, 0.02, 0.03, 0.04, 0.05), limits = c(0, 0.055))

mape_plot_rvs2

# MAPE boxplot plot

mape_bpxplot_rvs2 <- ggplot(long_res %>% filter(method=='RvS-2'), aes(x=as.factor(cstat), y=mape_est, fill=prev)) +

geom_hline(yintercept=c(0.01, 0.03, 0.05),linetype="dashed") +

geom_boxplot() + ylab("MAPE (2000 simulations - log scale")+ xlab("True c-statistic")+

theme_bw()+

theme(legend.position="bottom")+

labs(colour=long_res$prev) +

labs(fill="Prevalence", colour="Prevalence") + theme(text = element_text(size = 14))+

scale_y_continuous(trans='log10')

mape_bpxplot_rvs2

ggsave("plots development//rvs2_mape_boxplots.pdf", width=8, height=6)

ggsave("plots development//rvs2_mape_boxplots.png", width=8, height=6)

ggsave("plots development//figure_s3.png", width=8, height=6, dpi=600)

# MAPE size

size_plot_rvs2 <- res%>%ggplot(aes(cstat, n, group=method,col=method))+ geom_line(size=1) +

facet_wrap(~prev,labeller=prev_labeller) +

geom_text(aes(y = max(n)+1500, label=round(inflation,2)), color = "black", fontface = 2, size = 4.5) +

ylab("Sample size to achieve expected MAPE=prev/10 (log-scale)") + theme_bw()+

theme_bw() + theme(legend.position="bottom") +

xlab("True c-statistic") +

labs(shape="Method", colour="Method") + theme(text = element_text(size = 14)) +

scale_y_continuous(trans='log10', breaks=c(500, 700, 1000, 1400, 2000, 3000, 4000, 5000, 6230))

size_plot_rvs2

ggsave("plots development//rvs2_size_req_mape.pdf", width=13, height=6)

ggsave("plots development//rvs2_size_req_mape.png", width=13, height=6)

# MAPE EPV

epv_plot_rvs2 <- res%>%ggplot(aes(cstat, epv, group=method,col=method))+ geom_line(size=1) +

facet_wrap(~prev,labeller=prev_labeller) +

geom_text(aes(y = max(epv)+5, label=round(inflation,2)), color = "black", fontface = 2, size = 4.5) +

ylab("EPV to achieve expected MAPE=prevalence/10") +

scale_y_continuous(limits = c(0, 65)) +

theme_bw()+ theme(legend.position="bottom")+

xlab("True c-statistic") +

labs(shape="Method", colour="Method") + theme(text = element_text(size = 14))

epv_plot_rvs2

ggsave("plots development//rvs2_epv_req_mape.pdf", width=13, height=6)

ggsave("plots development//rvs2_epv_req_mape.png", width=13, height=6, dpi=600)

ggsave("plots development//figure_5.png", width=13, height=6, dpi=600)

wb <- createWorkbook()

addWorksheet(wb, "mape")

writeData(wb, 1, res)

addFilter(wb, 1, row = 1, cols = 1:ncol(res))

saveWorkbook(wb, file = "mape.xlsx", overwrite = TRUE)

View(res)

res_print <-cbind(res%>%filter(method=="By Simulation")%>%select(cstat, prev, n, epv, inflation),

res%>%filter(method=="RvS-2")%>%select(n, epv))

res_print <- arrange(res_print, prev, cstat)

names(res_print)=c("cstat", "prev","n_sim", "epv_sim","inflation","n_rvs2","epv_rvs2")

res_print<- select(res_print, prev, cstat, epv_rvs2, epv_sim, n_rvs2, n_sim, inflation)

res_print$n_rvs2 <- round(res_print$n_rvs2/10)*10

res_print$epv_rvs2 <- round(res_print$epv_rvs2,1 )

res_print$n_sim <- round(res_print$n_sim/10)*10

res_print$epv_sim <- round(res_print$epv_sim, 1)

View(res_print)

wb <- createWorkbook()

addWorksheet(wb, "Table MAPE")

writeData(wb, 1, res_print)

addFilter(wb, 1, row = 1, cols = 1:ncol(res_print))

saveWorkbook(wb, file = "Table_mape.xlsx", overwrite = TRUE)

saveWorkbook(wb, file = "table_s2.xlsx", overwrite = TRUE)

#################################################################################
